# Spatiotemporal Characterization of mTOR Kinase Activity Following Kainic Acid Induced Status Epilepticus and Analysis of Rat Brain Response to Chronic Rapamycin Treatment

**DOI:** 10.1371/journal.pone.0064455

**Published:** 2013-05-28

**Authors:** Matylda Macias, Magdalena Blazejczyk, Paulina Kazmierska, Bartosz Caban, Agnieszka Skalecka, Bartosz Tarkowski, Anna Rodo, Jan Konopacki, Jacek Jaworski

**Affiliations:** 1 Laboratory of Molecular and Cell Neurobiology, International Institute of Molecular and Cell Biology, Warsaw, Poland; 2 Department of Neurobiology, University of Lodz, Lodz, Poland; 3 Department of Pathology and Veterinary Diagnostics, Warsaw University of Life Sciences-SGGW, Warsaw, Poland; Universidade Federal do ABC, Brazil

## Abstract

Mammalian target of rapamycin (mTOR) is a protein kinase that senses nutrient availability, trophic factors support, cellular energy level, cellular stress, and neurotransmitters and adjusts cellular metabolism accordingly. Adequate mTOR activity is needed for development as well as proper physiology of mature neurons. Consequently, changes in mTOR activity are often observed in neuropathology. Recently, several groups reported that seizures increase mammalian target of rapamycin (mTOR) kinase activity, and such increased activity in genetic models can contribute to spontaneous seizures. However, the current knowledge about the spatiotemporal pattern of mTOR activation induced by proconvulsive agents is rather rudimentary. Also consequences of insufficient mTOR activity on a status epilepticus are poorly understood. Here, we systematically investigated these two issues. We showed that mTOR signaling was activated by kainic acid (KA)-induced status epilepticus through several brain areas, including the hippocampus and cortex as well as revealed two waves of mTOR activation: an early wave (2 h) that occurs in neurons and a late wave that predominantly occurs in astrocytes. Unexpectedly, we found that pretreatment with rapamycin, a potent mTOR inhibitor, gradually (*i*) sensitized animals to KA treatment and (*ii*) induced gross anatomical changes in the brain.

## Introduction

Mammalian target of rapamycin (mTOR) is a protein kinase that senses nutrient availability, trophic factors, cellular energy level, and cellular stress and adjusts cellular metabolism accordingly [1]. In neuronal cells, mTOR activity is additionally controlled by neurotransmitters [2]. mTOR plays several roles in neuronal development and plasticity [3]–[5]. It is involved in the control of neuronal survival [6]–[7], neurite growth [8]–[10], and synapse formation [9]. In adult animals it was shown that inappropriate mTOR activity had deleterious effects on learning and memory [5] or can disturb feeding behavior [11] as well as circadian rhythm [12]–[13].

The role of increased mTOR signaling has been proposed also in brain pathology. For example, genetic disorders characterized by mTOR hyperactivity (e.g., tuberous sclerosis) are often associated with a high probability of epilepsy [14]–[15]. In several animal models of epileptogenesis (e.g., kainic acid [KA]- or pilocarpine-induced status epilepticus), increased mTOR activity was biochemically proven [16]–[18]. Consequently, mTOR inhibitors have been proposed as a potential antiepileptogenic and antiepileptic treatment [19]–[20]. Rapamycin is one of the best known and widely used mTOR inhibitors. Rapamycin and its derivatives (e.g. RAD001) were recently tested in animal models of epilepsy for their potential to prevent various aspects of epileptogenesis [17]–[18], [21]–[26]. For example, Zeng et al. [17] and Buckamster et al. [18] found that the prolonged administration of rapamycin suppressed mossy fiber sprouting.

In contrast to relatively well studied consequences of mTOR upregulation in the brain, less is known regarding outcome of a chronic lack of mTOR activity. This is mostly due to the fact that mice lacking mTOR are not viable [27]–[28] and brain conditional knockout is currently being developed. Heterozygous mice are viable but do not have any gross changes in brain structure and distribution of variety of synaptic markers [29]. Also, under normal conditions their behavior appeared normal in several behavioral tests. Long-term application (10 weeks) of microcapsulated rapamycin with the food was able to decrease mTOR activity in the mouse brains but had no effects on performance in Morris water maze [30]. On the other hand, such treatment improved behavioral performance of transgenic mice mimicking Alzheimer disease (3x-Tg-AD) [30]. Also age-dependent cognitive problems in mice could be prevented in wild type mice with lifelong rapamycin administration [31]. This observation is in a striking contrast to the effects of administration of rapamycin intraperitoneally or directly to the structure, which had significant effects on behavior [32]–[34]. But thus far effects of chronic intraperitoneal rapamycin treatment on status epilepticus induction of wild type animals were not methodically evaluated.

Considering the potential importance of mTOR for brain physiology and pathology we systematically investigated two issues: (*i*) the spatiotemporal pattern of mTOR activation induced by proconvulsive agent KA in order to understand early events potentially contributing to epileptogenesis and (*ii*) effects of chronic rapamycin administration on brain morphology and response to KA. We found that mTOR signaling was activated by KA injection in several brain areas, including the hippocampus and cortex. We also found changes in the subcellular distribution of mTOR with KA treatment as well as discovered two waves of mTOR activation: an early wave (2 h) that occurs in neurons and a late wave that predominantly occurs in astrocytes. Furthermore, we found that long-term treatment with rapamycin gradually (*i*) sensitized animals to KA likely by changing threshold for KA-induced epileptic discharges and (*ii*) induced gross anatomical changes in the brain.

## Materials and Methods

### Antibodies and Reagents

The following antibodies were obtained from commercial sources: anti-mTOR (Cell Signaling Technology, Danvers, MA), anti-phospho-mTOR (Ser2448, P-mTOR; Cell Signaling Technology; for immunohistochemistry and Western blot analysis), anti-phospho-mTOR (Ser2448, P-mTOR; Signalway Antibody, Pearland, TX; for immunofluorescence analysis), anti-rpS6 (Cell Signaling Technology), anti-phospho-rpS6 (Ser235/236, P-S6; Cell Signaling Technology), anti-α tubulin (Sigma, St. Louis, MO), anti-NeuN (Millipore, Billerica, MA), anti-H3 (Sigma), anti-GFAP (Millipore), anti-RIP (Millipore), anti-pan-cadherin (Abcam, Cambridge, MA) and rabbit IgG (Santa Cruz Biotechnology, Dallas, TX). Anti-mouse or anti-rabbit secondary antibodies conjugated to Alexa Fluor 488, Alexa Fluor 568, horseradish peroxidase (HRP), IRDye 800CW, and IRDye 680 were obtained from Invitrogen (Eugene, OR), Jackson ImmunoResearch (West Grove, PA), and LI-COR (Lincoln, NE), respectively. Vectastain ABC detection kits were purchased from Vector Laboratories (Burlingame, CA). The following reagents were used for pharmacological treatments: rapamycin (LC Laboratories, Woburn, MA), kainic acid (Tocris Bioscience, Ellisville, MO), brain-derived neurotrophic factor (BDNF; Sigma), 6-cyano-7-nitroquinoxaline-2,3-dione (CNQX; Sigma), and tetrodotoxin (TTX; Alomone Labs, Jerusalem, Israel).

### Animals and Pharmacological Treatment

For the pharmacological experiments, adult, three months old male Wistar rats were used. For neuronal cultures, the brains were obtained from Wistar rat embryos on embryonic day 18 (E18). All of the procedures that involved animals and their care were conducted in conformity with the institutional guidelines of the First Local Ethics Committee in Warsaw (Decision No 843/2008), which are in compliance with the European Community Council Directive (86/609/EEC). All the described animal studies were approved by the appropriate ethics committee (i.e., First Local Ethics Committee in Warsaw, Decision No 843/2008). For the pharmacological experiments, the animals were divided into four groups: vehicle, KA, rapamycin, and rapamycin+KA. Rapamycin was initially dissolved in 100% ethanol and stored at −20°C. Immediately before the injection, rapamycin was diluted in a vehicle solution that contained 5% Tween 80 and 5% PEG 400 (low-molecular-weight grade of polyethylene glycol; Sigma) and injected intraperitoneally (i.p.; 10 mg/kg) three times per week for 1 week (short treatment) or 4 weeks (long treatment). A control group of rats was injected with vehicle solution that contained 5% Tween 80, 5% PEG 400, and 4% ethanol. Kainic acid (10 mg/kg) was delivered by i.p. injection. In the rapamycin+KA group, KA was injected 48 h after the last dose of rapamycin. The 48 h delay was used to avoid potentiating effects of immediate RA i.p. injections on KA-induced mTOR signaling [35]. Kainic acid-injected animals were observed for up to 6 h, and their behavior was digitally recorded (Movies 1 and 2). The rats were sacrificed 2 and 24 h following KA injection. Severity of seizures were assessed according to a modified Racine’s scale [36]: no response (0), twitching of face and ear, fictive scratching (1); myoclonic twitching and tremor (2), bilateral forelimb clonus with lordotic posture (3), forelimb clonus with reared posture (4), tonic-clonic seizure without postural control (5). Only animals, which displayed three episodes of generalized seizures (stage 3) where further analyzed.

### Neuronal Cell Culture and Pharmacological Treatment

Neuronal cells were obtained from the cortices of E18 rats as previously described [37]. After 2 weeks *in vitro*, the cells were first pretreated for 2 h with a mixture of 1 µM TTX and 100 µM CNQX to silence basal neuronal activity and then treated with either BDNF (0.1 ng/µl) or KA (100 µM) for 15 min.

### Semi-quantitive Immunohistochemistry (semi-Q-IHC)

For the immunohistochemical analysis of brain sections, the animals were lethally anesthetized with sodium pentobarbital and perfused transcardially first with phosphate-buffered saline (PBS) and next with 4% paraformaldehyde (PFA) in PBS. The brains were removed, postfixed in the same fixative for 2 h, and cryoprotected with 30% sucrose in 0.1 M phosphate buffer (PB) at 4°C. The brains were then frozen with pre-cooled heptane at approximately −30°C, placed on tissue holders, surrounded by the Jung tissue-freezing medium (Leica Microsystems, Wetzlar, Germany), and sectioned with a cryostat. Forty-micrometer free-floating coronal sections were collected in antifreeze medium (20% sucrose, 37.5% ethylene glycol in 0.05 M PB, pH 7.4) with 0.1 M sodium fluoride and kept at −20°C until use. When the enzymatic detection of the secondary antibodies was performed, the sections were first washed with PBS that contained 0.2% Triton X-100, pH 7.4 (PBST), incubated with a solution of 0.3% H_2_O_2_ in water for 20 min, again washed extensively in PBST, and incubated overnight at 4°C with primary antibody (anti-P-mTOR, 1∶200; anti-P-S6, 1∶200; anti-S6, 1∶500 in 1% normal goat serum [NGS; Vectastain ABC]) in PBST. Rabbit IgG diluted in 1% NGS was used as a negative control and final IgG concentration corresponded to the final dilution of anti-P-mTOR or anti-PS6 antibody. The next day, the sections were rinsed with PBST and incubated for 1 h at room temperature with biotinylated secondary antibody diluted in 1% NGS in PBST. Subsequently, after extensive washes with PBST, the sections were incubated for 1 h with AB complex that contained avidin-HRP conjugate (Vectastain ABC kit). Finally, the sections were washed with PBST and treated with 0.05% DAB (Sigma) and 0.01% H_2_O_2_. The reaction was terminated by the addition of PBST and subsequent washes with PBS. The sections were mounted on poly-L-lysine coated slides, dehydrated in ascending alcohol concentrations, cleared through xylene, and covered with DPX (Sigma).

Images of immunohistochemically stained brain sections were acquired under 10× magnification with the use of a Nikon Eclipse 80i microscope equipped with a monochromatic Evolution VF charge-coupled device camera (Media Cybernetics, Silver Spring, MD) and ImageProPlus software (Media Cybernetics). The light source power was stabilized during image acquisition to maintain the same illumination level for each imaging session. The settings of the camera and lamp were held constant. For brightfield microscopy, shading correction was applied. The brightness and contrast were adjusted to obtain images as close as possible to those observed directly under the microscope. The sections used for acquiring the images for the semi-quantitative analysis of P-mTOR and P-S6 were chosen (−2.56 to −3.60 mm from bregma) according to a rat brain atlas [38]. At least two brain sections for each rat were chosen for densitometric analyses. The subsequent regions of the brain were measured for assessing the mean optical density: pyramidal layer of *cornu ammonis* (CA) 1 and CA3, stratum oriens of CA1 and CA3, dentate gyrus (DG) granular layer, DG molecular layer, somatosensory cortex layers II–III, V and VI, layer I of piriform cortex, layer II of piriform cortex, amygdala (lateral amygdaloid nuclei: dorsolateral, ventrolateral and ventromedial part and basolateral nuclei: anterior and posterior part) and lateral hypothalamic area. In the hippocampus measurements for pyramidal and granular layers reflected signal from the cell bodies while those for stratum oriens and molecular layer was assigned to neuropil. Similar situation was for layer II (cell bodies) and I (neuropil) of piriform cortex. In case of somatosensory cortex and amygdala mean optical densities of the measured area could not be clearly assigned to either cell bodies or neuropil. Therefore, we measured mean optical density of (*i*) a whole outline region (total IR) and (*ii*) neuropil (done by sampling area between the cell bodies with 100 square pixel boxes). Six samples of non-cellular spaces were taken for each given region. The example outlines of each analyzed region are shown in relevant figures, where areas taken for cell body layer (hippocampus, piriform cortex) mean optical density measurements are surrounded with yellow line, areas taken for whole area (total, somatosensory cortex, amygdala) mean optical density measurements are surrounded with white line, while areas used for hippocampal and piriform cortex neuropil analysis are contoured with black line. Additionally, the densitometric signals of the background from the areas outside each section were collected and subtracted from the P-mTOR and P-S6 measurements. The data were then averaged per animal and compared between experimental groups. To assess optical density, ImageJ 1.44 software (National Institute of Mental Health, Bethesda, MD) was used.

### Immunofluorescence and Confocal Imaging

For double immunofluorescence labeling of brain sections, the sections were washed with PBST, blocked with 5% normal donkey serum in PBST, and incubated overnight at 4°C with anti-P-S6 antibody combined with anti-NeuN (1∶500), anti-GFAP (1∶1000), or anti-RIP (1∶1000) diluted in 1% normal donkey serum (Sigma) in PBST. Afterward, the sections were rinsed with PBST and labeled for 1 h at room temperature with the respective secondary antibodies conjugated to Alexa Fluor dyes diluted 1∶500 in 1% donkey normal serum in PBST. After several washes with PBS, the sections were mounted onto glass slides, air-dried, and covered with the VECTASHIELD Mounting Medium (Vector Laboratories).

For immunofluorescence staining, cultured neurons were fixed with 4% PFA and 4% sucrose in PBS for 10 min at room temperature. After fixation, the cells were rinsed with PBS and incubated for 15 min at 90°C with an antigen retrieval buffer (10 mM sodium citrate, 0.05% Tween 20, pH 6.0). After several PBS washes, the cells were first incubated with a blocking buffer (5% donkey normal serum, 0.3% Triton X-100 in PBS) at room temperature for at least 1 h and then overnight at 4°C with primary antibodies diluted in 1% BSA and 0.3% Triton X-100 in PBS. After incubation with the primary antibodies, the cells were washed three times with PBS and left for 1 h at room temperature with secondary antibodies conjugated to Alexa Fluor dyes diluted in 1% BSA and 0.3% Triton X-100 in PBS (1∶500). Prior to mounting with the VECTASHIELD Mounting Medium, the cells were washed with PBS.

Images of immunofluorescently stained brain sections and cultured cells were acquired using confocal microscopy. The confocal system consisted of a Zeiss LSM5 Exciter microscope equipped with lasers that produced light at 405, 488, and 568 nm wavelengths and was used for the acquisition of pictures of immunofluorescently labeled brain sections and cultured cortical neurons. Various objectives (20×/0.50, 40×/1.30) were used to scan the samples. A series of continuous optical sections at 1 µm intervals along the z-axis of a brain section or cell were scanned for all fluorescent signals and stored as a series of 1024×1024 pixel images. The laser power and image acquisition settings were kept constant.

### Terminal Deoxynucleotidyl Transferase dUTP Nick End Labeling (TUNEL) Assay

Dying cells were detected by TUNEL assay (In Situ Cell Death Detection Kit, TMR red, Roche Applied Science, Mannheim, Germany). Brain sections were permeabilized for 2 min on ice with permeabilization solution (0.1% Triton X-100, 1% sodium citrate), and incubated with labeling solution plus enzyme solution at 37°C for 1 hour (for negative control the enzyme solution was omitted). Next, the brain sections were washed three times with PBS-T and counterstained with Hochest 33258 dye and either anti-pan-cadherin or anti-NeuN antibody as described above.

### Preparation of Protein Extracts, Subcellular Fractionation, and Western Blot Analysis

For the biochemical studies, the brains were removed immediately after decapitation. From each brain, both hippocampi and the cerebral cortex were dissected, frozen on dry ice, and stored at −80°C until use. To extract brain proteins, the brain tissue was homogenized in tissue homogenization buffer (6 mM TRIS, pH 8, 150 mM NaCl_2_, 0.5 mM CaCl_2_, 1 mM MgCl_2_, 0.3% 3-[(3-cholamidopropyl)dimethylammonio]-1-propanesulfonate [CHAPS]), protease inhibitors (Roche, Indianapolis, IN), and phosphatase inhibitors (Sigma). For the nuclear *vs*. cytosolic P-mTOR studies, *in vitro* cultured cortical neurons [37] were first silenced as described above and next treated with KA (100 µM) for 15 min. The cells were then washed with PBS, followed by lysis and fractionation with the ProteoJET™ Cytoplasmic and Nuclear Protein Extraction Kit (Fermentas, Burlington, Canada). Protein concentrations in the obtained protein lysates were measured with the DC Protein Assay (Bio-Rad Laboratories, Hercules, CA).

The proteins were then analyzed by Western blot. After protein electrotransfer, the membranes were blocked for 1 h at room temperature in 5% nonfat dry milk in TBS-T and incubated overnight at 4°C with primary antibody against P-S6 (diluted 1∶500 in 5% BSA in TBS-T), S6 (diluted 1∶500–1∶1000 in 5% BSA in TBS-T), P-mTOR (diluted 1∶500 in 5% BSA in TBS-T), mTOR (diluted 1∶500–1∶1000 in 5% BSA in TBS-T), histone H3 (diluted 1∶5000 in nonfat dry milk in TBS-T), NeuN (diluted 1∶500 in 5% nonfat dry milk in TBS-T), and α-tubulin (diluted 1∶20000 in 5% nonfat dry milk in TBS-T). For enhanced chemiluminescent detection (ECL), the next day, the membranes were washed several times with TBS-T and incubated for 1 h with HRP-conjugated secondary antibody diluted in TBS-T that contained 5% nonfat dry milk. Finally, the membranes were washed with TBS-T, incubated for 1 min with ECL reagent, and immediately exposed to X-ray film. For fluorescence-based detection with the use of the Infrared Odyssey Imaging System (Li-Cor), after washes from the primary antibody, the membranes were incubated with IRDye-conjugated secondary antibodies diluted 1∶10000 in 5% nonfat dry milk in TBS-T. Afterward, the membranes were washed three times in TBS-T for 5 min. The membrane images were collected and analyzed with the Infrared Odyssey Imaging System.

### Morphometric Analysis of Brain Sections

For the morphometric analysis, hemispheric, hippocampal, and ventricular area Nissl-stained brain sections (−2.56 to −3.60 mm from bregma) were used. The analyzed regions were manually outlined and measured using ImageJ software.

### Electrophysiological Recordings

All the experiments described below were monitored and approved by the appropriate ethics committee (i.e., Local Ethics Committee in Lodz, permission no. 24/ŁB 547/2011; in accordance with the European Communities Council Directive of 24 November 1986). All the experiments were performed on 86 hippocampal formation (HPC) slices obtained from 12 male Wistar rats (150–250 g.) Each animal was anesthetized with halothane and decapitated. The brain was rapidly removed and placed in cold (3–5°C) and oxygenated (95% O_2_+5% CO_2_) artificial cerebrospinal fluid (ACSF; composition in mM: NaCl 121; KCl 5; CaCl_2_ 2.5; KH_2_PO_4_ 1.25; MgSO_4_ 1.3; NaHCO_3_ 26; glucose 10; Sigma Chemical Co., St. Louis, USA). ACSF was made fresh before each experiment, using prefiltered and deionized water. Transverse hippocampal slices (500 µm) were obtained from the HPC of two brain hemispheres using the tissue slicer (Stoelting, Wood Dale, IL). Hippocampal slices were incubated in oxygenated ACSF at about 20°C for 45 min after dissection. After this time, the slices were transferred into the gas-liquid interface recording chamber and maintained on nylon mesh, where they were continuously perfused with oxygenated and prewarmed (35°C) ACSF at a low (1 ml/min) flow rate for 45 min.

For the electrophysiological investigations the animals were divided into three experimental groups: acute treatment group with rapamycin solution, short treatment group with rapamycin solution, and long treatment group with rapamycin solution. Control recordings were conducted on HPC slices delivered from three groups of animals: acute treatment group with ethanol solution, short treatment group with vehicle solution, and long treatment group with vehicle solution. The acute treatment group was composed by HPC slices (delivered from non-pretreated animals) preincubated for 1.5 h in ACSF enriched with 100 nM ethanol (ACSF+ethanol, control) and in ACSF containing 100 nM rapamycin dissolved in ethanol (ACSF+ethanol+rapamycin). The short treatment group was composed by HPC slices delivered from animals pretreated for 1 week by vehicle solution (control) or rapamycin solution. The long treatment group was composed by HPC slices delivered from animals pretreated for 4 weeks by vehicle solution (control) or rapamycin solution. Please note that all slices of short- and long- treatment group were additionally treated as acute treatment group, i.e. they were preincubated for 1.5 h in ACSF enriched with 100 nM ethanol (ACSF+ethanol) or ACSF containing 100 nM rapamycin dissolved in ethanol (ACSF+ethanol+rapamycin). Details of the procedure of animals’ treatment was described in the section “*Animals and pharmacological treatment”*. Epileptiform discharges were evoked in HPC slices obtained from each group of animals by the application of KA in two doses: 0.05 µM and 0.5 µM. Recordings of the local field potential were performed with the use of glass recording electrodes (3–5 MΩ) made of Kwik-Fil capillaries (World Precision Instruments, Sarasota, FL). All recordings were performed from the CA3 region of HPC. The electrodes were positioned with the use of IVM micropositioner (Scientifica, Uckfield, UK). In all experiments, field potentials were recorded with respect to the ground. Signals were filtered (0.001–3 kHz, band pass) and amplified (x1000) using a P-511 preamplifier (Grass-Astromed, West Warwick, RI). The micro-EEG activity was displayed on a digital storage oscilloscope (TDS 3014, Tektronix, Beaverton, OR) and stored on a computer hard drive using data acquisition interface CED-1401 (Cambridge Electronic Design Ltd., Cambridge, UK). Computer analysis was conducted on 5 min fragments of EEG activity recorded between 20 and 50 min of the KA administration. Off-line spectral analysis of those fragments was conducted with the use of Spike 2.4 software (Cambridge Electronic Design Ltd.). The detailed analysis of the frequency of epileptiform discharges covered three 10 s fragments selected from each 5 min recordings. Mean values and standard deviations (SD) have been computed.

### Evans Blue Staining for Integrity of Blood–brain Barrier

For the blood–brain barrier permeability analysis, the animals were lethally anesthetized with sodium pentobarbital and perfused transcardially first with PBS and next with 1% Evans Blue (Sigma) followed again by PBS to wash out dye-excess from the brain vasculature. Subsequently the brains were removed, homogenized in 50% trichloric acid (1.3 ml/1 g. of tissue) and left in homogenization buffer for 96 h in 4°C. Next, homogenates were centrifuged for 10 min at 10000×g and absorbance of the supernatant was determined at 620 nm.

### Statistical Analysis

The results were analyzed with GraphPad Prism software (GraphPad, La Jolla, CA). The Mann-Whitney U-test was used for the statistical analysis.

## Results

### Kainic Acid Changes the Subcellular Distribution of mTOR Phosphorylated at Ser2448

Previous research provided evidence that different types of seizures can increase mTOR activity [17]–[18]. However, the detailed spatiotemporal analysis of the distribution of mTOR activity was not performed. Therefore, we began our research by comparing the spatiotemporal pattern of active mTOR expression in selected brain regions in control and KA-treated rats at various time-points after the drug injection. We immunohistochemically analyzed the expression pattern of mTOR phosphorylation at Ser2448 (P-mTOR), which is believed to reflect the activity of the kinase [39].

**Figure 1:**
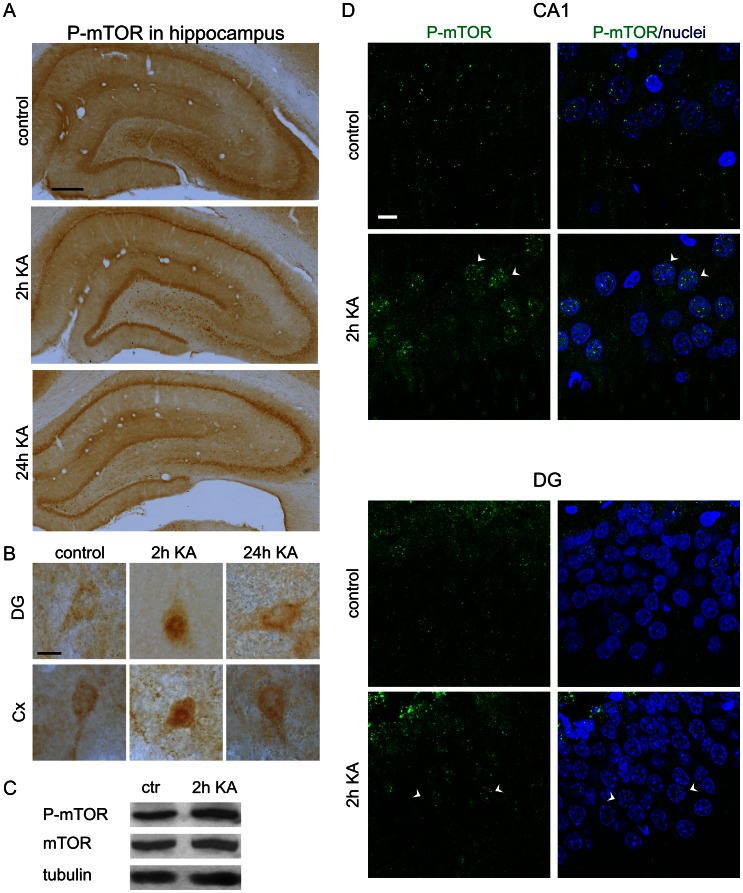
Kainic acid-induced changes in the subcellular distribution of mTOR phosphorylation at Ser2448. (***A***) Immunohistochemical analysis of P-mTOR expression in the hippocampus in control rats and in rats 2 and 24 h after kainic acid (KA)-induced status epilepticus. Scale bar = 200 µm. (***B***) Images of single cells of the dentate gyrus (DG) hilus (*upper panel*) and cortex *(lower panel*) of the animals described in *A*. Scale bar = 10 µm. (***C***) Representative results of Western blot analysis of hippocampal P-mTOR and mTOR levels in the control animals and in animals 2 h after KA injection. (***D***) Representative confocal images of double fluorescence staining with antibodies against P-mTOR (green) and nuclear dye Hoechst 33258 (blue) of the CA1 and DG regions of the hippocampus in control rats and in rats 2 h after KA administration. Arrowheads indicate double-stained nuclei. Scale bar = 10 µm.

**Figure 2:**
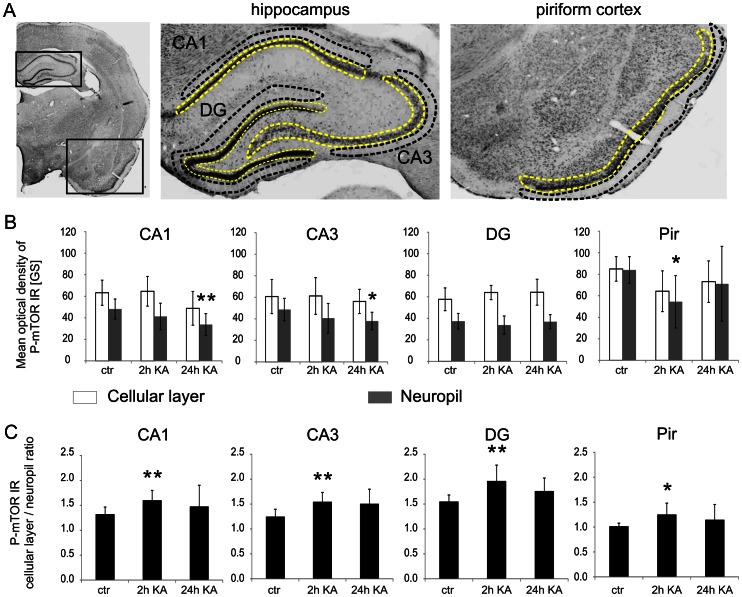
Quantitative analysis of kainic acid-induced changes in mTOR phosphorylation at Ser2448. (***A***) Representative micrographs of Nissl-stained brain sections with an outline of brain regions used for P-mTOR immunoreactivity (IR) semi-Q-IHC analysis. Areas of cell body layers for mean optical density measurements are surrounded with yellow line while areas used for hippocampal and piriform cortex neuropil analysis are contoured with black line. (***B***) Quantification of cellular layer and neuropil P-mTOR immunoreactivity in hippocampi and piriform cortex of control animals (*n* = 9) and animals treated with kainic acid (KA) and evaluated at 2 h (*n* = 6) and 24 h (*n* = 6) post KA. Error bars represent the standard deviation. Asterisks indicate significant changes in neuropil P-mTOR IR vs. control, *p<0.05, **p<0.01; Mann Whitney U-test. (***C***) Quantification of ratio of cellular layer P-mTOR IR to neuropil P-mTOR IR. Error bars represent the standard deviation. *p<0.05, **p<0.01; Mann Whitney U-test.

Visible P-mTOR immunoreactivity (IR) was observed in hippocampi and piriform cortex (Fig. 1 and Fig. S1) of control animals. In contrast, immunostaining was less visible in somatosensory cortex and amygdala (Fig. S1). No staining was observed when rabbit IgG was used instead of a primary antibody (Fig. S1). In the positively stained areas, both cell bodies and neuropil were IR positive. To analyze the P-mTOR immunohistochemistry results, we quantified P-mTOR IR by measuring the mean optical density within neuronal cell body and neuropil layers of hippocampus and piriform cortex (see Semi-Q-IHC in Materials and Methods and Fig. 2A). In control brains, P-mTOR immunostaining was uniformly distributed in the cell bodies and neuropil, both in CA1, CA3, and DG of the hippocampus and in piriform cortex (Fig. 1 and 2). Kainic acid treatment slightly changed the pattern of P-mTOR IR (Fig. 1A and 2B, C). However, the major change we observed in P-mTOR IR was rather its distribution than overall intensity (Fig. 2C) and we quantitatively confirmed increased ratio of cellular to neuropil P-mTOR IR for 2 h KA treatment (Fig. 2C). Our semi-Q-IHC analysis did not provide an ultimate answer whether KA changes mTOR phosphorylation because of differences in IR distribution. To address this issue, we performed a Western blot analysis of the protein extracts obtained from the brains of control animals and those treated with KA and assessed at 2 h and found only slight difference in either P-mTOR but not total mTOR levels in the hippocampus (Fig. 1C).

**Figure 3:**
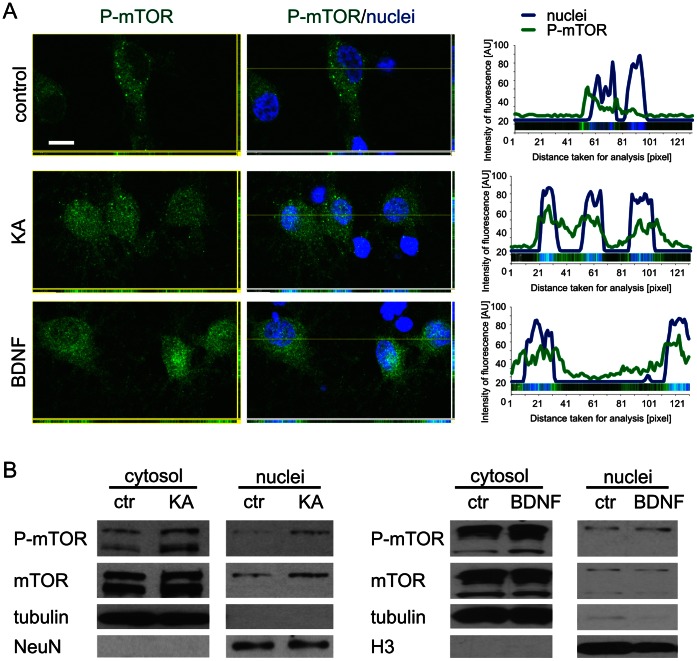
Kainic acid induces the nuclear presence of P-mTOR and total mTOR in neurons cultured *in vitro*. (***A***) Representative confocal images of cultured cortical neurons *in vitro* stained immunofluorescently for P-mTOR (green) and nuclear dye Hoechst 33258 (blue). Cortical neurons obtained from E18 rat embryos were cultured *in vitro* for 14 days. After the silencing of basal network activity (see Materials and Methods), the cells were treated for 15 min with either KA or BDNF. Single Z-sections are presented. The line graph shows the fluorescence intensity of P-mTOR (green) and Hoechst 33258 (blue) along the line running through the main image. The image of the analyzed area is placed at the bottom of each chart. AU, arbitrary units. Scale bar = 10 µm. (***B***) Western blot analysis of the subcellular fractions (cytosol and nuclei) obtained from cortical neurons cultured *in vitro* for 14 days and treated with either KA (*left panel*) or BDNF (*right panel*) as described in *A*.

In addition to changes in P-mTOR IR distribution between neuropil and cell bodies, one phenomenon was very consistent 2 h post-injection and did not occur either in control brains or at later time-points after KA treatment. At this time-point, we noticed the nuclear presence of P-mTOR in several neuron-like cells in the hippocampus and layer VI of the somatosensory cortex (Fig. 1B). Although such cells were very clearly visible because of the deeply dark-stained nuclei and relatively bright cytoplasm (Fig. 1B), we further confirmed the nuclear localization of P-mTOR in CA1 and DG cells by combined fluorescence staining with anti-P-mTOR antibody and nuclear dye Hoechst 33285 (Fig. 1D). Although the nuclear localization of mTOR was previously reported for non-neuronal cells [40]–[41], this has not been previously investigated in neurons. Therefore, to further investigate the subcellular distribution of P-mTOR with KA treatment, we performed additional experiments with neurons cultured *in vitro*. We treated embryonic cortical neurons with either 100 µM KA or 0.1 ng/µl BDNF, a well-known activator of the mTOR pathway in neurons [42], for 15 min. As shown in Fig. 3A, P-mTOR was virtually absent from the nuclear compartment under control conditions, but KA and BDNF induced its nuclear appearance. To further corroborate our observation using an independent method, we performed subcellular fractionation of the protein extracts obtained from *in vitro* cultured cortical neurons treated with KA as described above. As shown in Fig. 3B, both KA and BDNF increased the level of mTOR phosphorylation in the cytoplasmic as well as in nuclear fraction. The purity of the fractions was further confirmed using antibodies against NeuN or histone H3 (nuclear fraction) and tubulin (cytosolic fraction; Fig. 3B).

### Kainic Acid Induces Signaling Downstream of mTOR

**Figure 4:**
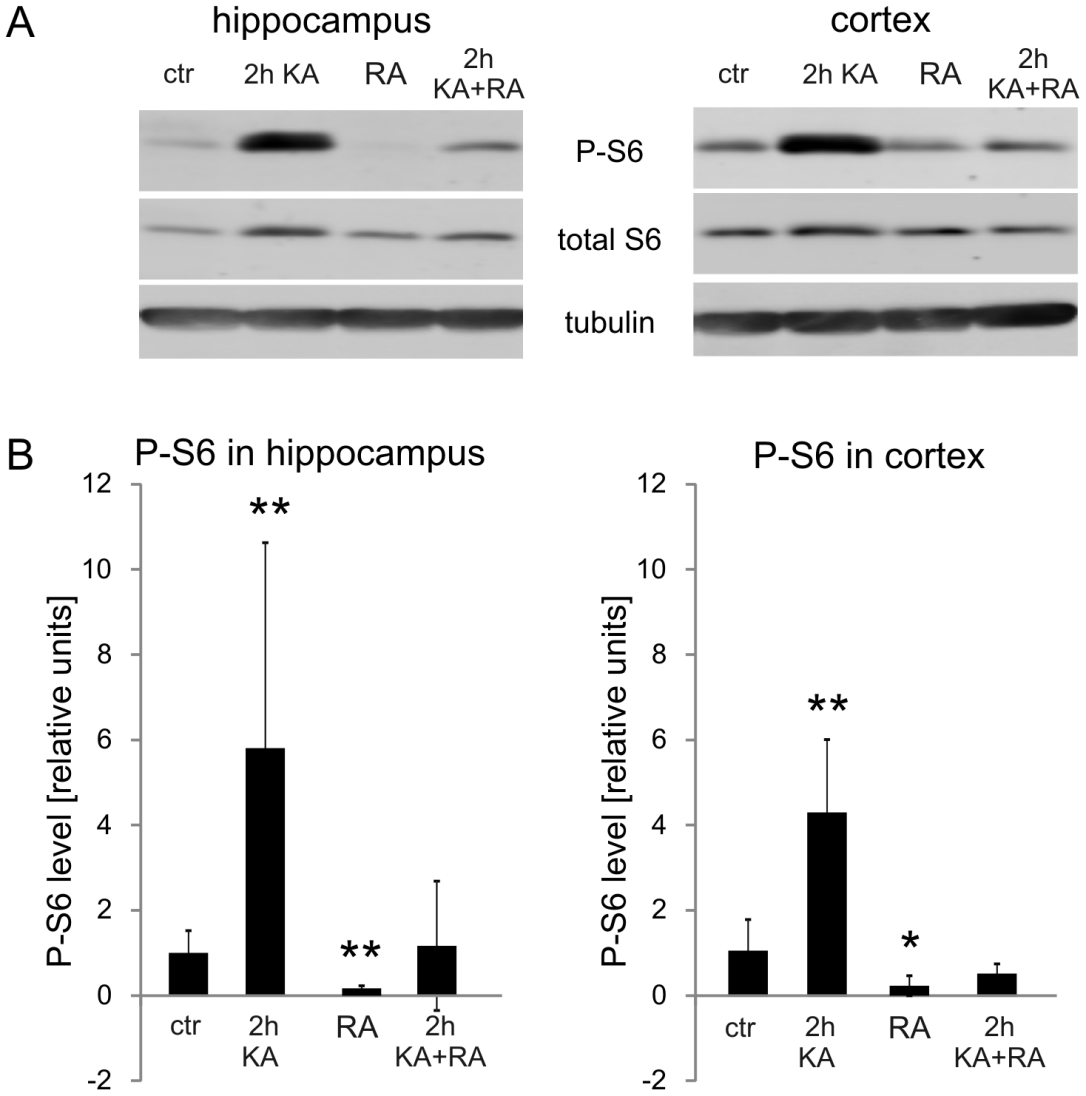
Phosphorylation of rpS6 at Ser235/236 increases with kainic acid-induced status epilepticus and depends on mTOR activity: a quantitative Western blot analysis. Quantitative Western blot analysis of phosphorylated rpS6 (P-S6) levels in the hippocampus and cortices in control (Ctr) animals (*n* = 5), animals that received kainic acid (KA) treatment and were evaluated at 2 h (*n* = 5), animals that received rapamycin (RA) treatment for 4 weeks (*n* = 6), and animals that received both KA and rapamycin treatment (*n* = 7). The graphs represent relative P-S6 levels normalized to tubulin. Error bars represent the standard deviation. *p<0.05, **p<0.01; Mann-Whitney U test.

**Figure 5:**
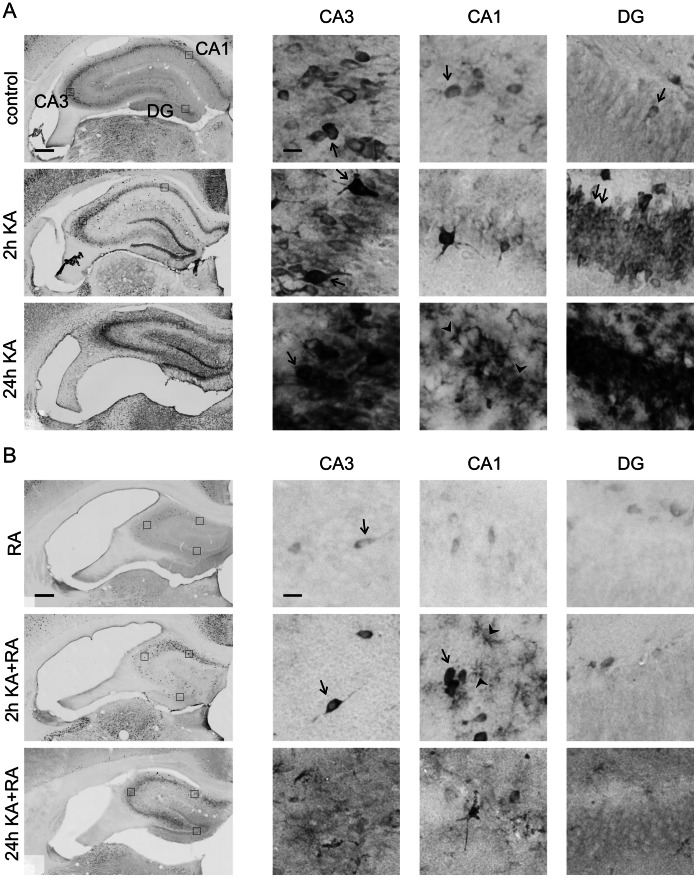
Phosphorylation of rpS6 at Ser235/236 in the hippocampus upon kainic acid-induced status epilepticus and rapamycin treatment. (***A***) Representative images of hippocampal sections immunohistochemically stained for P-S6 in control animals and in animals 2 and 24 h after kainic acid (KA) injection. Panels on the right represent higher-magnification of hippocampal regions boxed on the major view (left panels). (***B***) Representative images of hippocampal sections immunohistochemically stained for P-S6 in rapamycin- and rapamycin+KA-treated animals 2 and 24 h after KA treatment. Panels on the right represent higher-magnification of hippocampal regions boxed on the major view (left panel). RA – chronic rapamycin treatment. Arrows point to P-S6-positive neuronal-like cells. Arrowheads show glial-like P-S6-immunoreactive cells. Scale bar = 200 µm (left panel). Scale bar = 20 µm (right panel).

**Figure 6:**
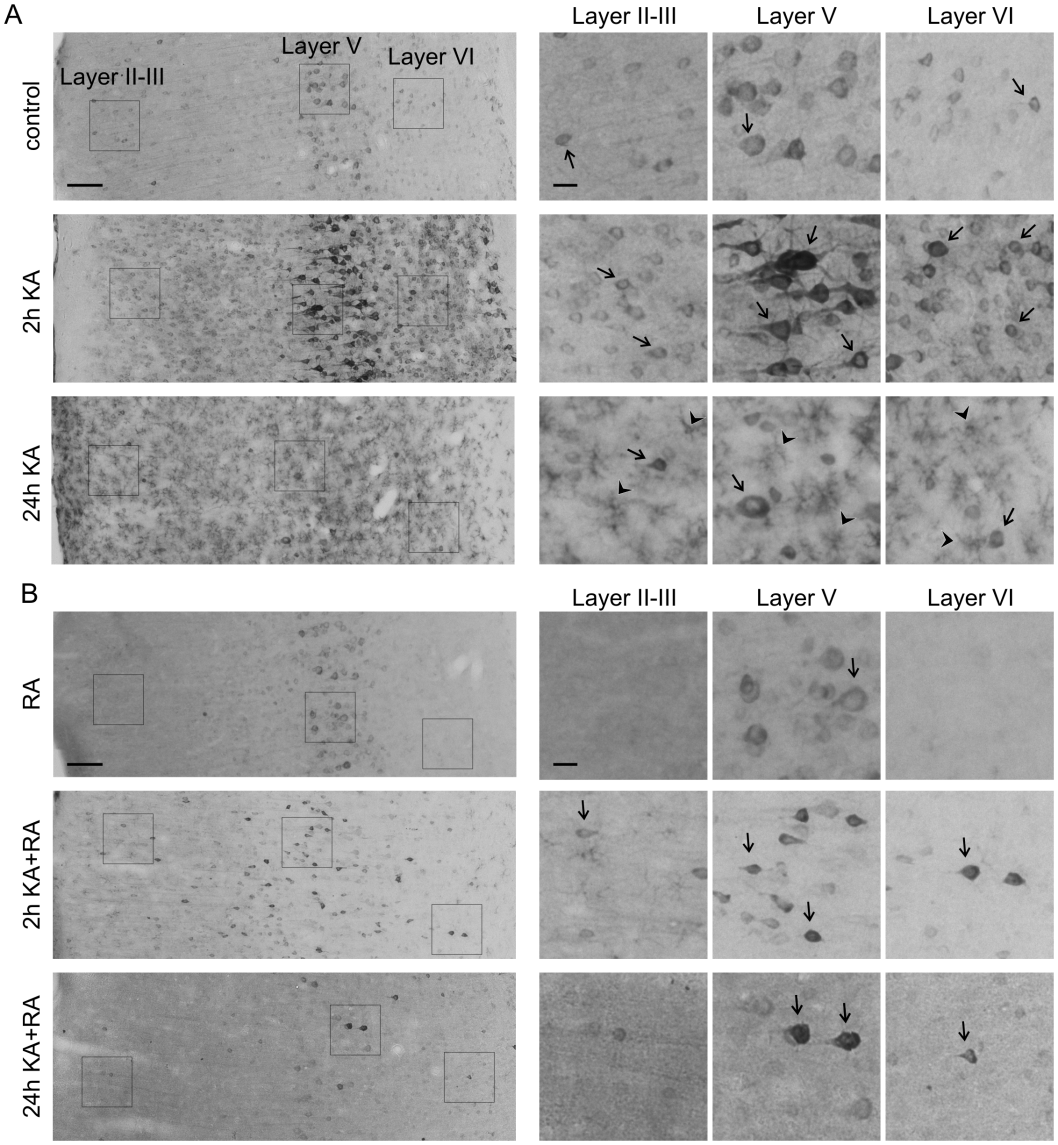
Phosphorylation of rpS6 at Ser235/236 in the somatosensory cortex upon kainic acid-induced status epilepticus and rapamycin treatment. (***A***) Representative images of somatosensory cortex sections immunohistochemically stained for P-S6 in control animals and in animals 2 and 24 h after kainic acid (KA) injection. Panels on the right represent higher-magnification of cortical layer regions boxed on the major view (left panels). (***B***) Representative images of somatosensory cortex sections immunohistochemically stained for P-S6 in rapamycin- and rapamycin+KA-treated animals 2 and 24 h after KA treatment. Panels on the right represent higher-magnification of cortical layer regions boxed on the major view (left panels). RA – chronic rapamycin treatment. Arrows indicate P-S6-positive neuronal-like cells. Arrowheads show glial-like P-S6-immunoreactive cells. Scale bar = 100 µm (left panel). Scale bar = 20 µm (right panel).

**Figure 7:**
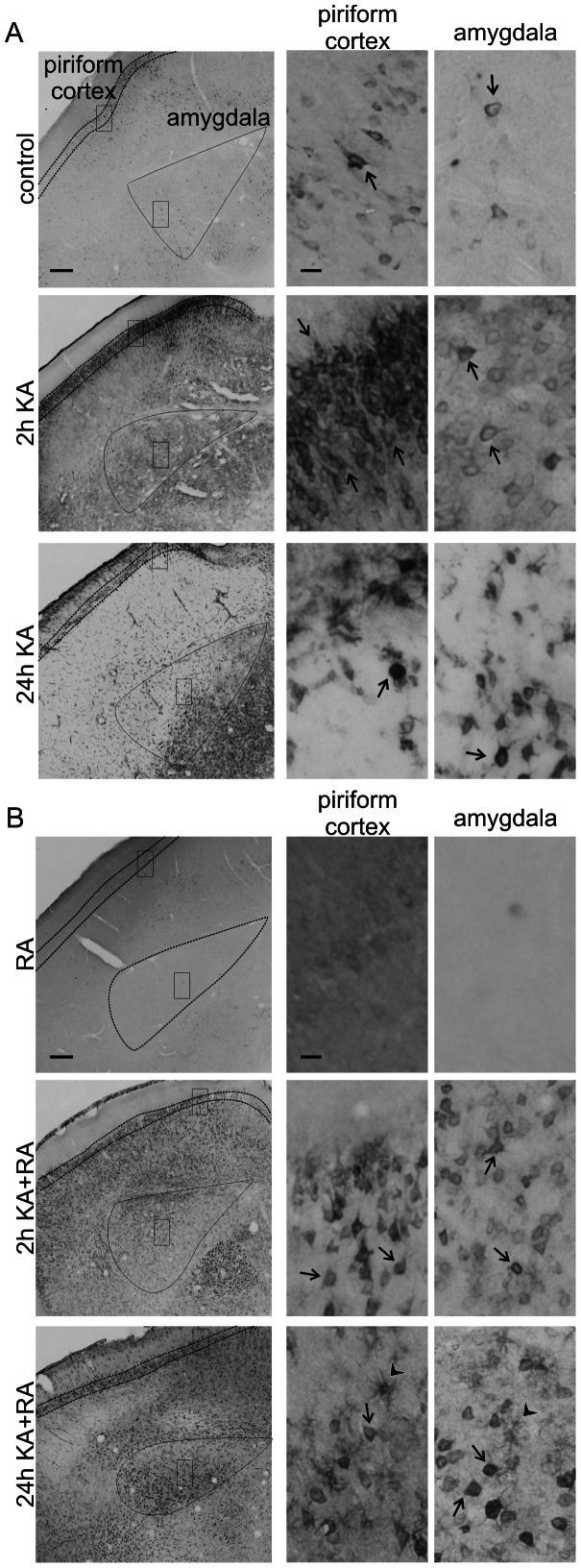
Phosphorylation of rpS6 at Ser235/236 in the piriform cortex and amygdala upon kainic acid-induced status epilepticus and rapamycin treatment. (***A***) Representative images of piriform cortex and amygdala sections immunohistochemically stained for P-S6 in control animals and in animals 2 and 24 h after KA injection. Dotted lines on the left panels outline piriform cortex and amygdala. Panels on the right represent higher-magnification of cortical and amygdalar regions boxed with the solid line on the major view (left panels). (***B***) Representative images of piriform cortex and amygdala sections immunohistochemically stained for P-S6 in rapamycin- and rapamycin+KA-treated animals 2 and 24 h after KA treatment. Dotted lines on the left panels outline piriform cortex and amygdala. Panels on the right represent higher-magnification of cortical and amygdalar regions boxed on the major view (left panels). RA – chronic rapamycin treatment. Arrows indicate P-S6-positive neuronal-like cells. Arrowheads show glial-like P-S6-immunoreactive cells. Scale bar = 200 µm (left panel). Scale bar = 20 µm (right panel).

**Figure 8:**
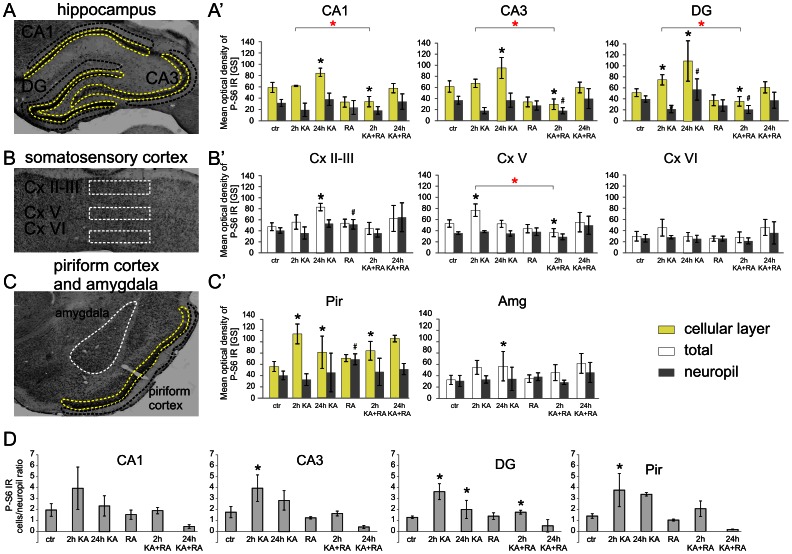
Quantitative analysis of kainic acid-induced changes in P-S6 phosphorylation at Ser235/236. (***A***) Representative micrographs of Nissl-stained brain sections with an outline of hippocampal regions used for quantitative P-S6 immunoreactivity (IR) analysis. Areas of hippocampal cell body layers for mean optical density measurements are surrounded with yellow line while areas used for hippocampal neuropil analysis are contoured with black line. (***A’***) Quantification of a mean P-S6 IR of cellular layers and neuropil of the hippocampi of control animals (*n* = 8), animals treated with rapamycin (RA) (*n* = 5), animals treated with kainic acid (KA) and evaluated at 2 h (*n* = 4), animals treated with KA and evaluated at 24 h (*n* = 6), animals treated with KA and rapamycin and evaluated at 2 h (*n* = 4), and animals treated with KA and rapamycin and evaluated at 24 h (*n* = 2). (***B***) Representative micrographs of Nissl-stained brain sections with an outline of somatosensory cortex regions used for quantitative P-S6 IR analysis. Areas of particular cortical layers used for whole region (total) mean optical density measurements are surrounded with white line (***B’***) Quantification of a mean P-S6 IR of the whole outlined region (total) and neuropil in the somatosensory cortex of animals treated as in *A’* (***C***) Representative micrographs of Nissl-stained brain sections with an outline of piriform cortex and amygdala regions used for semi-quantitative P-S6 IR analysis. Area of piriform cortex cell body layer for mean optical density measurements is surrounded with yellow line while area used for piriform cortex neuropil analysis is contoured with black line. Area of amygdala used for whole region (total) mean optical density measurements are surrounded with white line (***C’***) Quantification of a mean P-S6 IR of cellular layer of piriform cortex, whole outlined region of amygdala and neuropil (piriform cortex or amygdala) of animals treated as in *A’*. Error bars represent the standard deviation. Black asterisks indicate significant changes in cellular layer or total P-S6 IR vs. control, *p<0.05; Mann Whitney U-test. Red asterisks indicate significant changes in cellular layer or total P-S6 IR vehicle vs. rapamycin treated, *p<0.05; Mann Whitney U-test. Crosses indicate significant changes in neuropil P-S6 IR vs. control, ^#^ *p*<0.05; Mann Whitney U-test. (***D***) Quantification of ratio of cell layer P-S6 IR to neuropil P-S6 IR in the hippocampi and piriform cortex of animals treated as in *A’*. Error bars represent the standard deviation. Asterisks indicate significant changes vs. control. *p<0.05; Mann Whitney U-test.

Our analysis of P-mTOR did not provide the ultimate answer if mTOR activity is indeed changed after KA-induced status epilepticus. Therefore, for analyzing mTOR kinase activity, we tested the phosphorylation of ribosomal protein (rp) S6 (Ser 235/236; P-S6), an indirect mTOR target and a frequently used indicator of mTOR activity [17], [24], [43]. We first verified with quantitative Western blot (Q-WB) that indeed we can reproduce the previously described immediate increase in P-S6 [17]. As shown in Fig. 4, low levels of P-S6 were already detectable under basal conditions and significantly increased 2 h post-KA injection both in the hippocampus and cortex. We then analyzed the P-S6 activation pattern in more detail across different parts of the brain using semi-Q-IHC (see Materials and Methods). The presence of P-S6 in control brains was detected in cell bodies and neuropil in all tested brain areas, including the hippocampus, cerebral cortex, amygdala (Fig. 5A, 6A, 7A), and numerous thalamic and hypothalamic nuclei (Fig. S1), regions previously studied in context of neuronal activation by KA [44]–[46]. Such staining was not observed when brain sections were incubated with rabbit IgG (Fig. S1). Everywhere, the P-S6 IR was either equally distributed between cell bodies and neuropil or slightly higher in cell bodies (Fig. 8). Most of the P-S6-positive cells were neuron-like, but some glia-like cells were also present. Among the hippocampal regions, P-S6-immunoreactive cells were present in the CA1 and CA3 (Fig. 5A and 8A’). Less P-S6-immunoreactive cells were found in the DG (Fig. 5A, and 8A’). In the somatosensory cortex, cells in layer V were visibly stained for P-S6, whereas very few labeled ones were found in the other cortical layers (Fig. 6A and 8B’). Piriform cortex was stained similar to DG (Fig. 7 and 8C’). In the amygdala, P-S6 IR was weak (Fig. 7 and 8C’). Two hours after KA treatment, a visible enhancement of P-S6 IR was found in granular layer of the DG, cortical layer V and layer II of piriform cortex (Fig. 5A, 6A, 7A and 8). At the same time, neuropil staining of CA3 and DG slightly decreased (Fig. 5A, 8A’). No significant changes were observed in neuropil of layer V and layer I of piriform cortex (Fig. 5A, 6A, 7A and 8). At 24 h post-KA increased P-S6 IR was observed in cell body layers of CA1, CA3 and DG. Also was increase in total P-S6 IR of cortical layers II and III. At the same time P-S6 IR of cell bodies in the piriform cortex and total IR of cortical layer V returned almost to control levels (Fig. 5A, 6A, 7A and 8). However, P-S6 IR in layer I of the piriform cortex decreased (Fig. 7A, 8C’). KA also significantly increased P-S6 IR. Similarly to analysis performed for P-mTOR IR we verified if there was a shift in distribution of P-S6 IR between neuropil containing layers of hippocampus and piriform cortex and those enriched in neuronal cell bodies. As shown in Fig. 8D, KA injection resulted in significant shift of P-S6 IR towards cell body layers at 2 h in CA3, DG and piriform cortex. Notably, the IR of total S6 did not change dramatically at all tested time-points (Fig. S2).

It should be however noted that serines 235 and 236 of rpS6 can be phosphorylated by multiple kinases [47]–[49] and therefore mTOR-dependence of S235/236 phosphorylation should be tested under conditions of mTOR inhibition. Thus, to confirm that the observed changes in the level and distribution of P-S6 upon KA administration can indeed be attributed to mTOR activity and further investigate the role of mTOR activation in KA-induced seizures, we included in studies described above groups of rats chronically (4 weeks) treated with rapamycin. The Western blot analysis of brain protein extracts obtained from rapamycin-treated rats revealed decrease in the levels of P-S6 in the hippocampus and cerebral cortex compared with vehicle-treated control animals both under basal conditions as well as 2 h post-KA (Fig. 4). The effects of rapamycin were further checked with semi-Q-IHC. As shown in Figs. 5, 6, 7, 8, rapamycin quite effectively lowered levels of P-S6 in all investigated areas of hippocampi but in the other tested areas rapamycin was less effective under basal conditions (e.g. somatosensory cortex, amygdala, piriform cortex). Upon KA injection rapamycin significantly blocked P-S6 induction in hippocampi and layer V of somatosensory cortex (Fig. 5, 6, 7, 8).

### Activation of rpS6 in Neurons and Astrocytes

**Figure 9:**
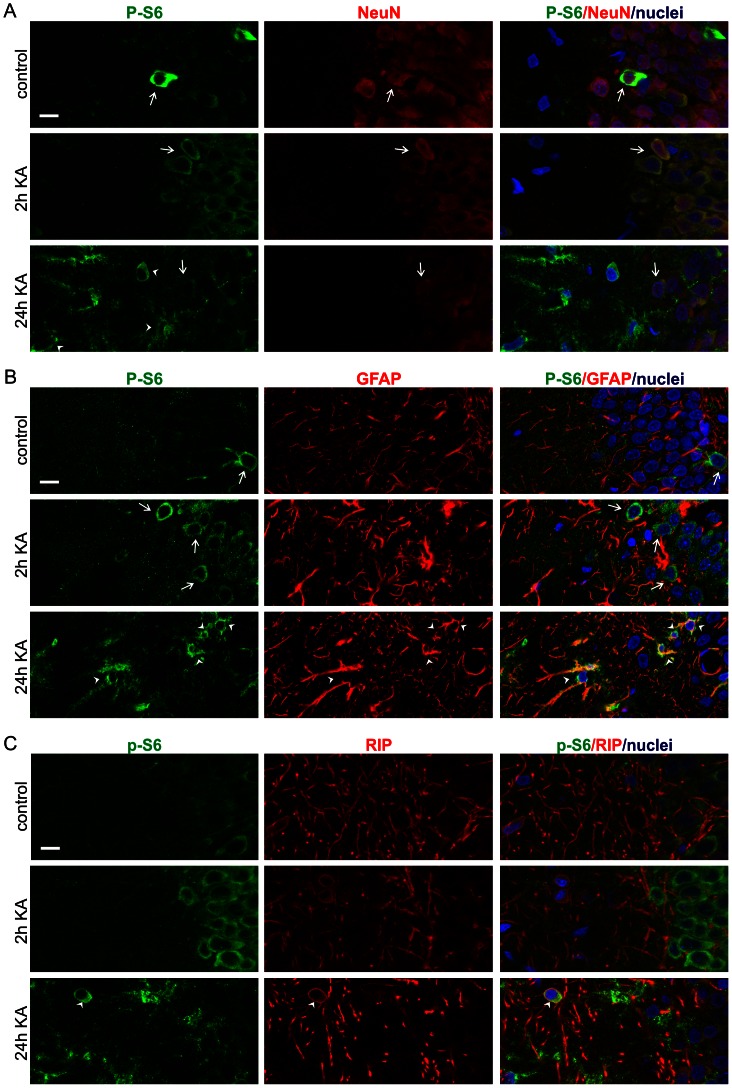
Kainic acid induces two cell type-specific waves of rpS6 phosphorylation. (***A***) Representative confocal images of DG sections immunofluorescently stained for P-S6 (green) and neuronal marker NeuN (red) in control animals and in animals that received kainic acid (KA) treatment and were evaluated at 2 and 24 h. (***B***) Representative confocal images of DG sections immunofluorescently stained for P-S6 (green) and astrocytic marker GFAP (red) in control animals and in animals that received KA treatment and were evaluated at 2 and 24 h. (***C***) Representative confocal images of DG sections immunofluorescently stained for P-S6 (green) and oligodendrocytic marker RIP (red) in control animals and in animals that received KA treatment and were evaluated at 2 and 24 h. Arrows and arrowheads indicate neuronal and glial cells, respectively. Scale bar = 10 µm.

Upon closer inspection of P-S6 IR in the hippocampus at later time-points (e.g., 24 h post-KA treatment), its upregulation could be assigned to a large population of glial-like cells that were not stained at earlier time-points and in control brains (Fig. 5). Such a shift from neuronal-like to glial-like P-S6 IR was even clearer in all layers of the cerebral cortex where neuronal-like profiles were rarely scattered, whereas glial-like cells densely covered the tissue (Fig. 6 and 7). In the amygdala, glial-like profiles were also present (Fig. 7). To confirm the shift in the type of P-S6-immunoreactive cells over time, we performed double immunofluorescence staining with anti-P-S6 antibody and antibodies against specific markers of neurons (NeuN), astrocytes (glial fibrillary acidic protein [GFAP]), and oligodendrocytes (RIP antigen). As shown in Fig. 9, in the DG of control animals, P-S6-positive cells also stained for NeuN but not GFAP or RIP. The increase in P-S6 immunofluorescence observed 2 h after KA injection was strictly related to the NeuN-positive population (Fig. 9A). However, P-S6 immunofluorescence detected 24 h after KA injection was also very abundant in GFAP-positive glial cells (Fig. 9B). We observed few P-S6 immunoreactive oligodendrocytes at this time-point (Fig. 9C). Additionally, we noted more intense GFAP labeling in all of the examined brain areas, which is frequently observed following status epilepticus [50]–[52]. Notably, rapamycin treatment weakened not only the neuronal but also the glial presence of P-S6 IR and the intensity of GFAP IR after KA (Fig. S3). No visible changes in the intensity of RIP or NeuN immunostaining were found at any of the tested time-points.

### Rapamycin Sensitizes Animals to KA-treatment and Evokes Morphological Changes in the Rat Brain

**Figure 10:**
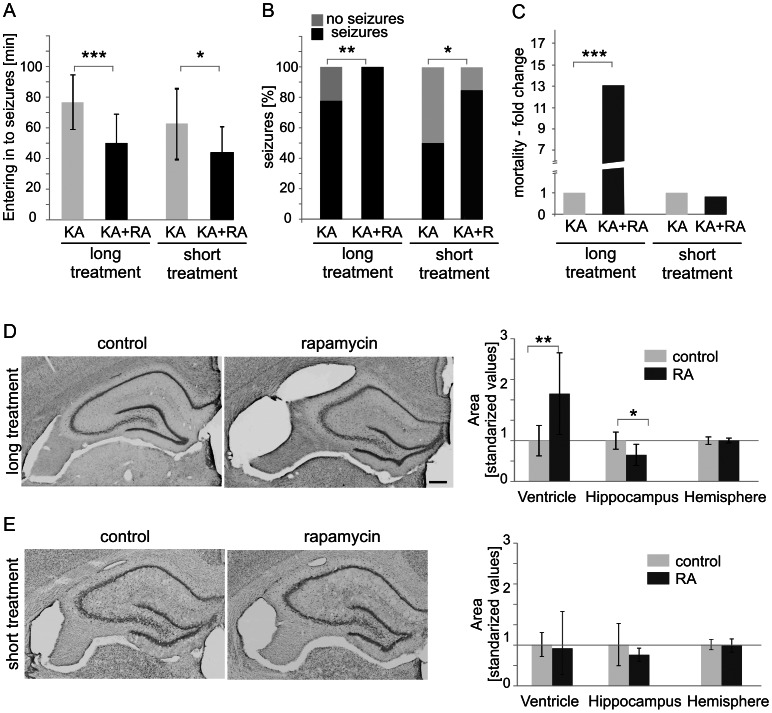
Chronic rapamycin treatment influences seizure susceptibility, KA-induced mortality and gross brain morphology. (***A, B, C***) Analysis of mean latency from kainic acid (KA) injection to stage 3 seizures (*B*) percentage of animals which developed seizures (*C*) and mortality, induced by KA in rats treated for either one week (short treatment) or four weeks (long treatment) with vehicle (short treatment: *n* = 20; long treatment: *n* = 14) or RA (short treatment: *n* = 20; long treatment: *n* = 36). Error bars represent the standard deviation. *p<0.05; **p<0.01; ***p<0.001; Mann Whitney U-test. (***D***) Representative images of Nissl-stained hippocampal sections in animals after long treatment with vehicle (*n* = 7) or RA (*n* = 8). Scale bar = 200 µm. (***E*** *)* Analysis of changes in the hippocampus, ventricular and entire hemisphere areas in rats treated as in *D*. Error bars represent the standard deviation. *p<0.05, **p<0.01; Mann Whitney U-test. (***F***) Representative images of Nissl-stained hippocampal sections in animals after short treatment with vehicle (*n* = 8) or RA (*n* = 6). Scale bar = 200 µm. (***G*** *)* Analysis of changes in the hippocampus, ventricular and entire hemisphere areas in rats treated as in *F*. Error bars represent the standard deviation.

In course of our experiments described above, we noticed different behavioral response to KA of animals pretreated for 4 weeks with rapamycin. Because previous work showed no effects of 3-day pretreatment with rapamycin on KA-induced status epilepticus [17], in a second part of our work we decided to follow our initial observation and analyze in detail effects of 1 week (short treatment) and 4 weeks (long treatment) rapamycin pretreatments on status epilepticus. As summarized in Fig. 10, we found that animals treated with rapamycin either for 1 (see Fig. S4 for biochemical confirmation of treatment effectiveness) or 4 weeks were more sensitive to KA. These animals reached stage 3 seizures earlier than KA-only-treated rats (Fig. 10A). Moreover, significantly more of the rapamycin+KA-treated animals developed seizures (stage 3 or higher) when compared with rats which received KA alone (Fig. 10B). Additionally, the seizures were followed by greater mortality in the group of animals treated with KA after 4-week rapamycin treatment (Fig. 10C).

**Figure 11:**
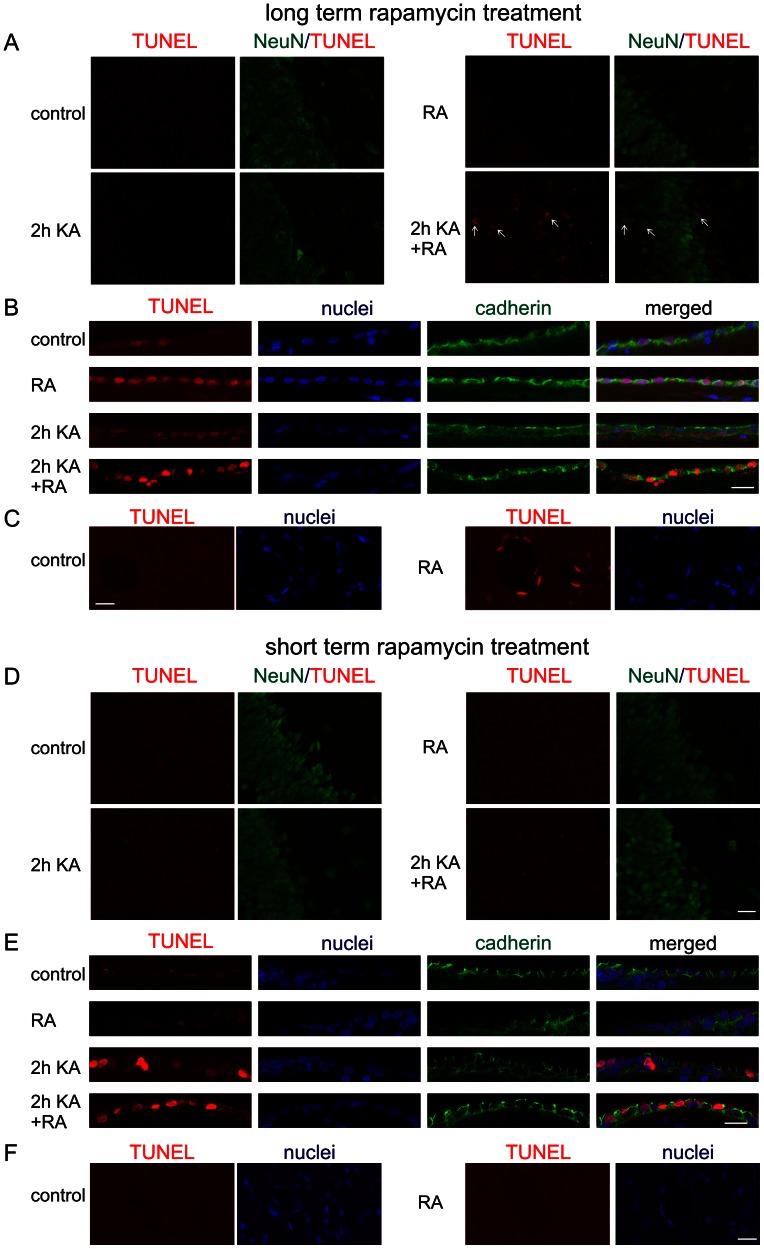
Long-term treatment with rapamycin increases ependymal but not neuronal cell death. (***A***) Representative confocal images of hippocampal sections stained for presence of apoptotic cells (TUNEL staining, red) and neuronal marker NeuN (green), obtained from control animals, animals treated with rapamycin for 4 weeks (long treatment), animals treated with KA and evaluated at 2 h, animals treated with KA and rapamycin and evaluated at 2 h. Arrows point dually labeled neurons. Scale bar = 20 µm. (***B***) Representative confocal images of ependymal cells surrounding lateral ventricle, stained for presence of apoptotic cells (TUNEL staining, red) and counterstained with cadherin antibody to visualize ependymal cells (green) and nuclear dye Hoechst 33258 (blue), obtained from animals treated as in *A*. Scale bar = 20 µm. (***C***) Representative confocal images of ependymal cells surrounding brain blood vessels, stained for presence of apoptotic cells (TUNEL staining, red) and counterstained with nuclear dye Hoechst 33258 (blue), obtained from animals treated as in *G*. Scale bar = 20 µm. (***D***) Representative confocal images of hippocampal sections stained for presence of apoptotic cells (TUNEL staining, red) and neuronal marker NeuN (green), obtained from control animals, animals treated with rapamycin for 1 week (short treatment), animals treated with KA and evaluated at 2 h, animals treated with KA and rapamycin and evaluated at 2 h. Arrows point dually labeled neurons. Scale bar = 20 µm. (***E***) Representative confocal images of ependymal cells surrounding lateral ventricle, stained for presence of apoptotic cells (TUNEL staining, red) and counterstained with cadherin antibody to visualize ependymal cells green) and nuclear dye Hoechst 33258 (blue), obtained from animals treated as in *D*. Scale bar = 20 µm. (***F***) Representative confocal images of ependymal cells surrounding brain blood vessels, stained for presence of apoptotic cells (TUNEL staining, red) and counterstained with nuclear dye Hoechst 33258 (blue), obtained from animals treated as in *D*. Scale bar = 20 µm.

In search for potential reasons of chronic rapamycin-induced sensitization to KA we performed pathomorphological analysis. We found that long, but not short, treatment with rapamycin caused brain morphological changes, i.e. shrunk the hippocampi and enlarged the ventricles (Fig. 10D, E). At the same time the total area of the analyzed brain sections was very similar between experimental groups (Fig. 10D). Brain anatomy changes were rather not an effect of systemic failure in response to long rapamycin treatment since we did not observe significant differences between groups in pathology of hearts and kidneys. This anatomical difference induced by long-term rapamycin treatment was also not an effect of increased neuronal cell death since we did not observe increased neuronal apoptosis with TUNEL staining in hippocampi of either long or short rapamycin pretreated animals (Fig. 11A, D). However, long treatment increased hippocampal cell death of NeuN positive cells in response to KA (Fig. 11A). Curiously, several ependymal cells of brain ventricles were TUNEL positive upon long term rapamycin treatment (Fig. 11B). We noticed also some TUNEL positive epithelial cells of the brain blood vessels under such condition (Fig. 11C). To verify leakiness of blood-brain barrier we analyzed its permeability to Evans Blue dye. As shown in Fig. S5, long treatment with rapamycin indeed increased amount of Evans Blue extracted with trichloric acid from the brain homogenates, however difference did not reach statistical significance due to high variation between individual animals. There were no signs of epithelial cell death or increased blood brain barrier permeability in brains of animals after short rapamycin treatment (Fig. 11E, F and Fig. S5). Thus, we concluded that hippocampal shrinkage and ependymal cell death observed after long-treatment with rapamycin are unlikely to contribute to increased sensitization of animals to KA, but could contribute to increased KA-induced mortality.

### Chronic but not Acute Rapamycin Treatment Increases KA-induced Epileptic Discharges in Acute Brain Slices

**Figure 12:**
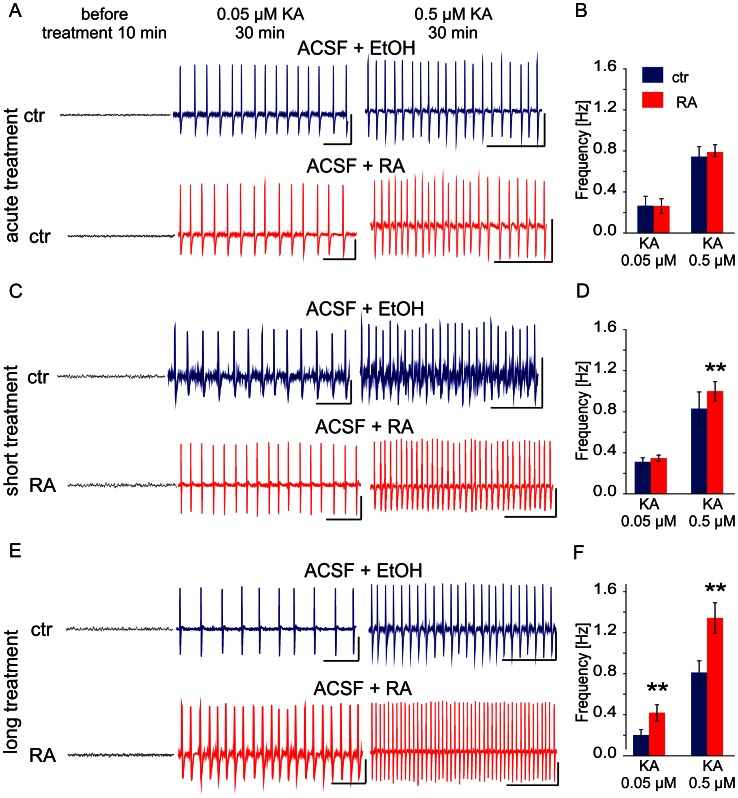
Chronic treatment with rapamycin gradually increases frequency of kainic acid induced epileptiform field activity in acute hippocampal slices. (***A***) Analogue examples of epileptiform activity recorded in 30 min after kainic acid (KA) administration (0.05 µM, 0.5 µM) in CA3c region of HPC slices delivered from non-injected animals (acute treatment). Prior to (1.5 h) and during recordings slices were treated either with vehicle (ACSF+EtOH, marked blue) or with 100 nM rapamycin (ACSF+RA, marked red). Calibration: 10 s and 1000 µV. (***B***) Quantification of the frequency (x ± SD) of KA induced epileptiform field activity in hippocampal formation slices treated with vechicle (*n* = 5 for 0.05 µM KA; *n* = 6 for 0.5 µM KA) or rapamycin (*n* = 6 for 0.05 µM KA; *n* = 5 for 0.5 µM KA). (***C***) Analogue examples of epileptiform activity recorded in 30 min after KA administration (0.05 µM, 0.5 µM) in CA3c region of HPC slices delivered from animals treated for 1 week (short treatment) with vehicle (ACSF+EtOH, marked blue) or RA (ACSF+RA, marked red). Prior to (1.5 h) and during recordings slices were treated either with vehicle or with 100 nM rapamycin (RA). Calibration: 10 s and 1000 µV. (***D***) Quantification of the effect of short rapamycin treatment on frequency (x ± SD) of KA induced epileptiform field activity in hippocampal formation slices taken from control (*n* = 8 for 0.05 µM KA; *n* = 9 for 0.5 µM KA) and short time rapamycin treated animals (*n* = 7 for 0.05 µM KA; *n* = 8 for 0.5 µM KA), **p<0.01; Mann-Whitney U test. (***E***) Analogue examples of epileptiform activity recorded in 30 min after KA administration (0.05 µM, 0.5 µM) in CA3c region of HPC slices delivered from animals treated for 4 weeks (long treatment) with vehicle (ACSF+EtOH, marked blue) or RA (ACSF+RA, marked red). Prior to (1.5 h) and during recordings slices were treated either with vehicle or with 100 nM rapamycin (RA). Calibration: 10 s and 1000 µV. (***F***) Quantification of the effect of long rapamycin treatment on frequency (x ± SD) of KA-induced epileptiform field activity in hippocampal formation slices taken from control (*n* = 8 for 0.05 µM KA; *n* = 8 for 0.5 µM KA) and short time rapamycin treated animals (*n* = 9 for 0.05 µM KA; *n* = 7 for 0.5 µM KA). **p<0.01; Mann-Whitney U test.

Since neither morphological changes nor increased cell death could account for increased KA sensitivity caused by short rapamycin treatment we tested the hypothesis that mTOR inhibition leads to decreased excitation threshold and higher epileptic discharge rate in response to KA. Towards this end, we analyzed epileptic discharges between 20 and 50 min after KA administration generated in the hippocampal slices, obtained from rapamycin pretreated animals (one or four weeks). As controls we used slices from animals treated with vehicle. Additionally we tested effects of acute exposure of slices delivered from vehicle-treated rats to rapamycin. The frequency [Hz] of induced discharges was used as the parameter, which mirrors the severity of epileptiform activity [53]–[54]. The epileptiform discharges were induced by two doses of KA: 0.05 µM and 0.5 µM. As shown in Fig. 12A, the acute treatment with rapamycin did not modulate the frequency of KA induced epileptiform activity. Short treatment with rapamycin (1 week) increased the frequency of discharges when compared to control only in a presence of a higher dose of 0.5 µM KA (control: 0.83±0.16 Hz; short rapamycin treatment: 1.00±0.09 Hz, *p*<0.01) (Fig. 12B). Finally, long treatment with rapamycin (4 weeks) increased the frequency of epileptiform discharges in response to both KA doses when compared to non-rapamycin treated slices. Specifically, in a presence of 0.05 µM KA the frequency of epileptiform discharges increased from 0.22±0.05 Hz to 0.45±0.08 Hz (*p*<0.01), while in a presence of a 0.5 µM KA, the frequency of epileptiform discharges increased from 0.86±0.12 Hz to 1.43 Hz ±0.16 Hz (*p*<0.01) (Fig. 12C). These data suggest that chronic rapamycin treatment gradually decreases threshold for KA induced epileptiform discharges and increases their frequency.

## Discussion

The principal aims of the present study were to establish mTOR activity patterns in different cell types and brain regions following KA-induced status epilepticus and investigate the effects of chronic, systemic rapamycin treatment on susceptibility to status epilepticus. We showed that KA treatment activated mTOR signaling in several brain areas (e.g., hippocampus, cortex). Curiously, we provided evidence of an increased nuclear presence of phosphorylated mTOR in neurons 2 h after KA administration. Moreover, we observed a time-dependent switch in the type of cells with active mTOR signaling, from neurons to glia, approximately 24 h after the KA injections. With regard to the second aim, we showed that chronic systemic rapamycin treatment, which inhibited mTOR signaling in hippocampi and cortex gradually led to facilitation of KA-induced status epilepticus and epileptic discharges as well as induced gross morphological changes in the brain.

Using both, biochemical and immunohistochemical approaches, we found increased mTOR kinase signaling through cortical areas and the hippocampus already 2 h after KA that lasted until 24 h. This observation is consistent with previously published results [16]–[17]. The added value of our work, however, is the information about brain structure identity, brain cell identity, and subcellular mTOR distribution provided by the semi-Q-IHC approach used herein. We were able to reveal two novel aspects of mTOR signaling with KA treatment: (*i*) increased IR for P-mTOR in the nuclei of several neurons 2 h after KA injection and (*ii*) the existence of the time-dependent cellular specificity of mTOR response to seizures. In the present study, we analyzed the detailed pattern of P-mTOR (S2448) IR in rat brains following KA-induced status epilepticus. Although, functional role of this phosphorylation remains unknown it is believed to be characteristic for intact and active mTORC1 [39]. In control brains, P-mTOR IR was rather diffuse and present in neuropil, and the cell bodies. This changed, however, 2 h after KA treatment, in which P-mTOR IR was enriched in cell bodies (e.g., hippocampus and piriform cortex). Furthermore, several neuronal cell nuclei stained positively for P-mTOR throughout the cerebral cortex and hippocampus. Because we report the presence of P-mTOR in neuronal nuclei for the first time its meaning remains unknown but work done thus far on non-neuronal cell lines, suggests that mTOR activity in the nucleus is linked to control of all three RNA Polymerases [55]–[57]. Another intriguing observation derived from our immunohistochemical analysis was that we observed two waves of mTOR activity-dependent P-S6 expression after KA treatment: an initial wave in neurons and a later wave in glial cells. Similar observation was recently done by Sha et al. [58] who showed that intrahippocampal KA infusion induces P-S6 firstly in neurons (6 h post KA) and next in astrocytes (5 days post KA). Here we show that mTOR pathway activation in astrocytes occurs much earlier that could be anticipated from previous report. Sha et al. [58] and our observations raise two important issues (*1*) why mTOR is activated in neurons and glia with different time course and (*2*) whether role of increased P-S6 in neurons and glia of KA treated animals is epileptogenic or protective. In neurons mTOR signaling is known to be activated by both neurotransmitters, including glutamate and trophic factors, e.g. BDNF [2], [4], [59], release of which is induced by KA [60]. Thus, most likely the first neuronal wave of P-S6 is a response to such stimuli. On the other hand, the second, glial wave of P-S6 IR occurs at 24 h time point, which corresponds to KA-induced neuronal death and astrogliosis [61]–[62]. What exactly induces mTOR in astrocytes after KA is not known but it could be either one of an astriogliosis triggers or secondary mediators released by activated glial cells. For example, mTOR activation in spinal cord astrocytes was driven by EGF and was needed for their activation [63]. On the other hand, in cultured cortical astrocytes mTOR responded to proinflamatory cytokines [64], that are released by activated glia. Our data, show that rapamycin, which prevented KA-triggered increase in GFAP immunofluorescence also inhibited P-S6 IR in astrocytes. This suggests that mTOR activation is rather induced by initial signals leading to glial activation.

What is a role for KA-driven activation of mTOR pathway? Both neuronal and glial cells participate in the pathophysiology of seizures and epileptogenesis [50]–[51]. For example, neurons either die or undergo plastic changes that can lead to aberrant neuronal connectivity. Indeed, the immediate activation of mTOR by KA leads to neuronal cell death [17]. In contrast, the delayed wave that occurs days after the seizures contributes to aberrant sprouting [17]. Thus, so far evidence supports mTOR epileptogenic functions in neurons. As mentioned above another outcome of KA-induced seizures is reactive astrocytic gliosis and mTOR likely plays an important role in this process. Astrocytic transformation increases neuronal excitability [62], [65]–[66]. In fact observations by Sha et al. [58] that P-S6 can be observed in reactive astrocytes even 6 weeks after KA in mice as well as in sclerotic hippocampi of epileptic patients may suggests its role in epileptogenic changes. However, reactive astrocytes express proteoglycans and other molecules that inhibit axon growth and may attenuate aberrant axon sprouting, which appears to be a protective role of astrocytes at later stages of epileptogenesis [67]–[68]. Thus, the roles of the different waves of mTOR activity in epileptogenesis require further study.

Rapamycin is currently considered potential anti-epileptogenic drug [19]–[20], when applied after initial stimulus, although data regarding this issue are conflicting [17], [23]–[24], [69]. In some cases systemic rapamycin treatment in a rat model of temporal lobe epilepsy was reported to suppress the frequency of acquired seizures, cell death, and axonal sprouting [17], [23]. At the same time, Zeng et al. [17] showed that rapamycin injection prior to treatment with KA had no effect on the severity of status epilepticus induced by proconvulsant injection. However, in the present study, we found that already one-week rapamycin pretreatment accelerated proconvulsive effects of KA. Four-week pretreatment enhanced these effects even further as well as elevated KA-induced mortality. Our study and the previous study by Wong group [17] have major technical difference that may explain the discrepancy. In the previous studies, rapamycin pretreatment was relatively short (3 days) [17] Thus, our work does not conflict with the previous reports but rather provides new knowledge regarding combined effects of chronically low mTOR activity and proconvulsive stimulation. In this context, an interesting question is why chronic rapamycin treatment enhanced the effects of KA. One potential answer is that chronic rapamycin treatment leads to changes in blood-brain barrier (BBB) permeability which is considered important factor in epilepsy [70]. But, our data does not support such mechanism since mild effects of rapamycin on survival of blood vessel ependymal cells and BBB permeability were observed only after 4-week pretreatment. Another explanation for increased susceptibility to KA in rats chronically treated with rapamycin could be decreased threshold of neuronal excitability. In fact, our electrophysiological data obtained with use of acute slices from rapamycin pretreated animals support such mechanism. We showed that chronic pretreatment of animals with rapamycin increased severity to KA-induced epileptic discharges in slices (Fig. 12). What is more, with longer pretreatment lower doses of KA were sufficient for induction of discharges (Fig. 12). At the same time acute pretreatment of slices with rapamycin had no effect on KA induced discharges, what fits well with observation of Zeng et al. [17] described above. While we did not provide molecular mechanism for rapamycin driven decrease in neuronal excitation threshold, it has been reported that rapamycin application to cultured *in vitro* neurons can change dramatically expression of neuronal and glial glutamate transporters and several potassium channels [71].

Unclear is how rapamycin affects brain anatomy and why similar changes were not previously reported. Hippocampal size reduction often is a result of hippocampal cell death. However, using TUNEL staining we did not detect increased cell death in the hippocampal formation neither after 1 week nor 4 weeks of rapamycin treatment. On the other hand, our results show increased cell death of ventricle ependymal cells. This could result in less efficient circulation of cerebrospinal fluid and hydrocephalus, and favor the hypothesis that mTOR inhibition affects hippocampal size due to increased pressure of oversized ventricles. Further experiments will clarify this issue. With regard to lack of previous reports of rapamycin effects on brain anatomy, one explanation may be that long-term rapamycin treatment was used previously in genetically modified mice that already had enhanced mTOR signaling [e.g., PTEN knockout, TSC1+/−; 21–22,72]. Thus, rapamycin may have reestablished brain homeostasis. A similar explanation can be made for animals that received rapamycin treatment after pharmacologically induced seizures. However, work done by Oddo laboratory [30]–[31] do not fall into the same category and cannot be explained that way. In their work Caccamo et al. [30] and Majumder et al. [31] treated mice chronically with rapamycin and did not observe any negative impact of such treatment on cognitive functions. Although they did not evaluate gross brain morphology, it is very unlikely that such changes as reported herein would leave learning abilities intact. In our opinion, the most probable explanation of discrepancies between our work and studies of Caccamo et al. [30] and Majumder et al. [31] is substantial difference in experimental design in three aspects, animal species used for tests (mice vs. rat), route of rapamycin administration (oral vs. intraperitoneal) and time scale of experiments. First two dissimilarities may lead to either different final concentration of a drug in the brain tissue or non-comparable dynamics of inhibition (gradual vs. relatively fast). With regard to the third one, it was recently shown that after initial phase (up to 6 weeks) of deteriorative effects of rapamycin on several aspects of mouse metabolism, such effects disappeared after further treatment (up to 20 weeks) [73]. In fact, our experiments were performed in “deterioration” phase while the Oddo’s group in recovery one. Further studies are needed in this regard but fact of brain recovery during chronic rapamycin treatment is potentially very important for all studies on mTOR regarding either brain physiology or pathology.

## Supporting Information

Figure S1 **Immunoreactivity of anti-P-mTOR, anti-P-S6 and rabbit IgG in selected rat brain regions.** Representative images of layers II, III and V of somatosensory cortex, dentate gyrus (DG) of hippocampus, amygdala, piriform cortex and nuclei of thalamus (Rt - reticular thalamic nucleus; ventral posterolateral thalamic nucleus) of control animals. Scale bar = 50 µm.
(TIF)

Figure S2 **Kainic acid treatment does not change significantly levels of total rpS6 in the hippocampus and somatosensory cortex of rats.** Representative images of hippocampus (left panel) and somatosensory cortex (right panel) sections immunohistochemically stained for total rpS6 of control animals and of animals 2 and 24 h after kainic acid (KA) injection. Scale bar = 200 µm (left panel). Scale bar = 50 µm (right panel).
(TIF)

Figure S3 **Kainic acid induces and rapamycin prevents increase in GFAP expression and in P-S6 phosphorylation in astrocytes. (** ***A*** **)** Representative of dentate gyrus sections immunofluorescently stained for P-S6 (green) and astrocytic marker GFAP (red) in control animals and in animals that received kainic acid (KA) treatment and were evaluated at 2 and 24 h. **(** ***B*** **)** Representative images of DG sections immunofluorescently stained for P-S6 (green) and astrocytic marker GFAP (red) in chronically rapamycin (RA)- and RA+KA-treated animals 2 and 24 h after KA treatment. Scale bar = 20 µm.
(TIF)

Figure S4 **One week treatment with rapamycin lowers basal and KA-induced phosphorylation of rpS6 at Ser235/236 in rat cortex.** Western blot analysis of phosphorylated rpS6 (P-S6) levels in the cortices in control (Ctr) animals, animals that received kainic acid (KA) treatment and were evaluated at 2 h, animals that received rapamycin (RA) treatment for 1 week, and animals that received both KA and rapamycin treatment.
(TIF)

Figure S5 **Analysis of effects of chronic rapamycin (RA) treatment on permeability of blood brain barrier with Evans Blue (EB) dye.** Quantification of absorbance of supernatants obtained from brain lysates of control and rapamycin treated rats after trichloric acid precipitation (see Materials and Methods for details). Short treatment = 1 week rapamycin treatment (3 doses per week). Long treatment = 4 weeks rapamycin (3 doses per week). Control animals received vehicle.
(TIF)

Movie S1 **Severity of seizures** **in kainic acid treated rats.** The movie shows behavior of rats (104 sec. sequence), 80 minute after KA injection, when the first visible signs of response to drug occurred. The grade of seizures is coded with different colors of arrows pointing to seizing animals, i.e.: myoclonic twitching and tremor (green arrows), bilateral forelimb clonus with lordotic posture (blue arrows).
(MP4)

Movie S2 **Severity of kainic acid induced seizures** **in rapamycin pretreated rats.** The movie shows behavior of rats (158 sec. sequence), starting at 50 min. after KA injection, when the first visible signs of response to drug occurred. The grade of seizures is coded with different colors of arrows pointing to seizing animals, i.e.: myoclonic twitching and tremor (green arrows), bilateral forelimb clonus with lordotic posture (blue arrows), forelimb clonus with reared posture (pink arrows), tonic-clonic seizure without postural control (red arrows); death of animal (black arrow).
(MP4)
