# Supplementary figures and images for: Spatiotemporal Characterization of mTOR Kinase Activity Following Kainic Acid Induced Status Epilepticus and Analysis of Rat Brain Response to Chronic Rapamycin Treatment
Source: PLoS One. 2013 May 28;8(5):e64455. doi: 10.1371/journal.pone.0064455 (PMC3665782; doi:10.1371/journal.pone.0064455)

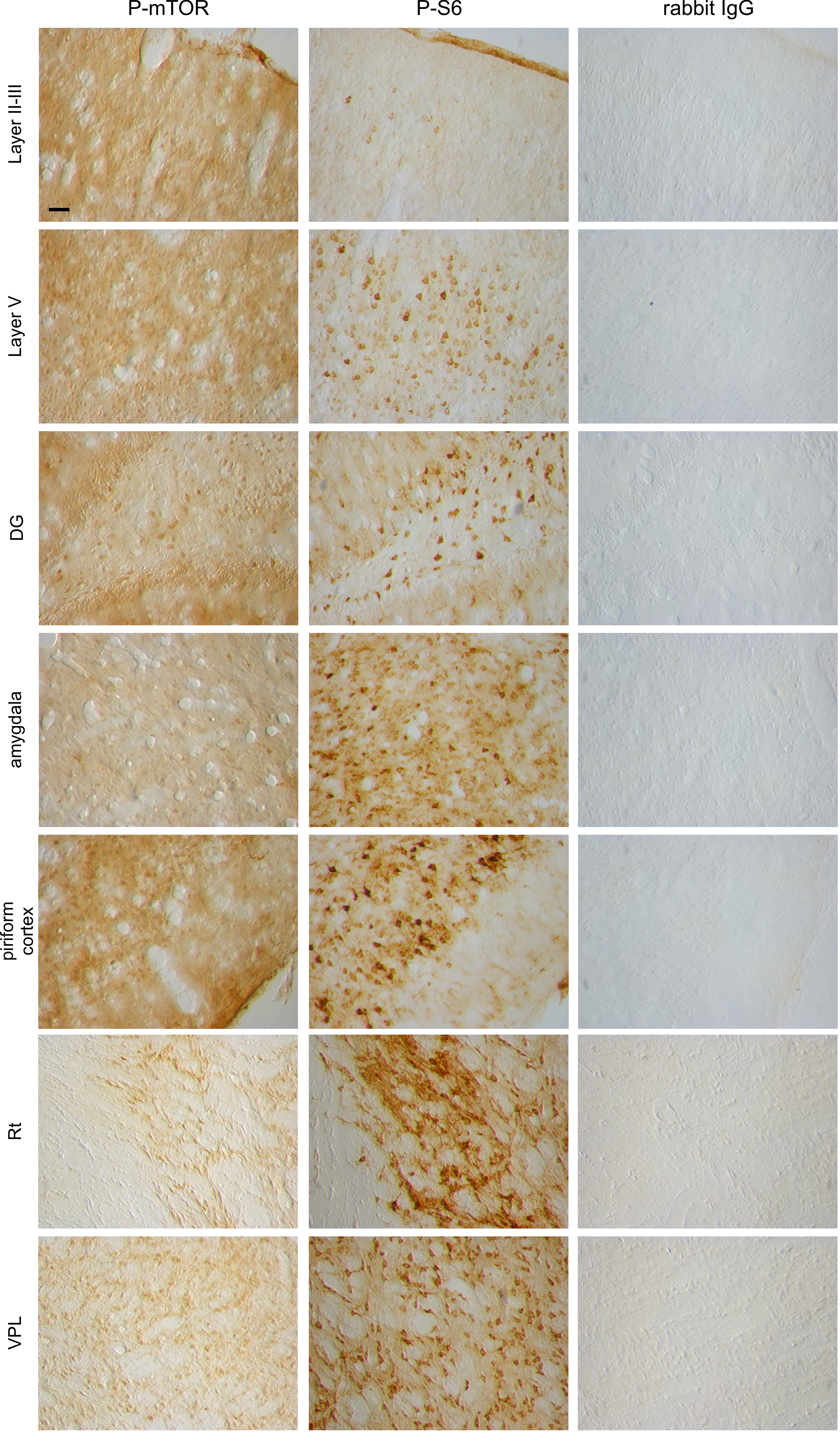

Supplement: Figure S1 — Immunoreactivity of anti-P-mTOR, anti-P-S6 and rabbit IgG in selected rat brain regions. Representative images of layers II, III and V of somatosensory cortex, dentate gyrus (DG) of hippocampus, amygdala, piriform cortex and nuclei of thalamus (Rt - reticular thalamic nucleus; ventral posterolateral thalamic nucleus) of control animals. Scale bar = 50 µm. (TIF) [file pone.0064455.s001.tif]

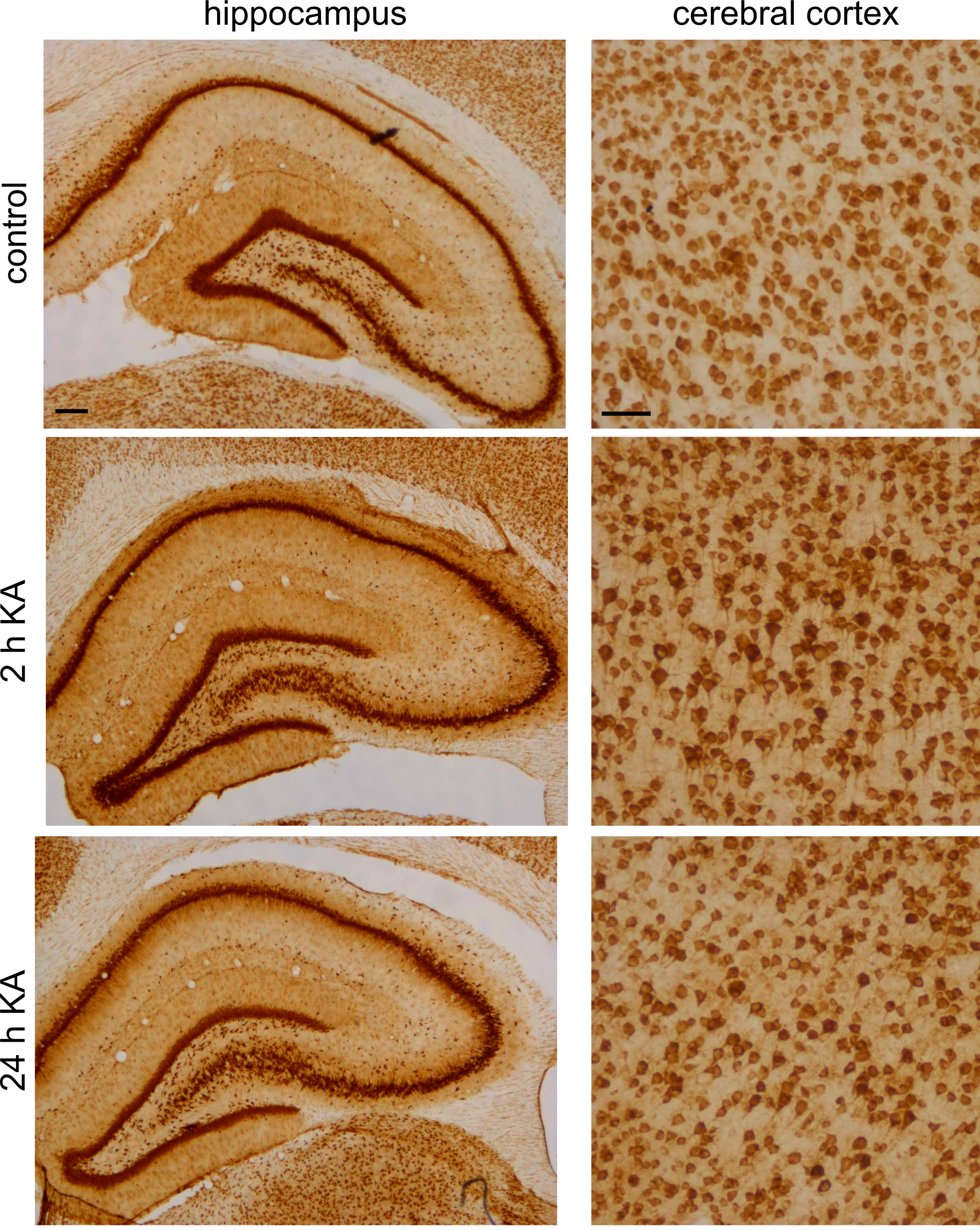

Supplement: Figure S2 — Kainic acid treatment does not change significantly levels of total rpS6 in the hippocampus and somatosensory cortex of rats. Representative images of hippocampus (left panel) and somatosensory cortex (right panel) sections immunohistochemically stained for total rpS6 of control animals and of animals 2 and 24 h after kainic acid (KA) injection. Scale bar = 200 µm (left panel). Scale bar = 50 µm (right panel). (TIF) [file pone.0064455.s002.tif]

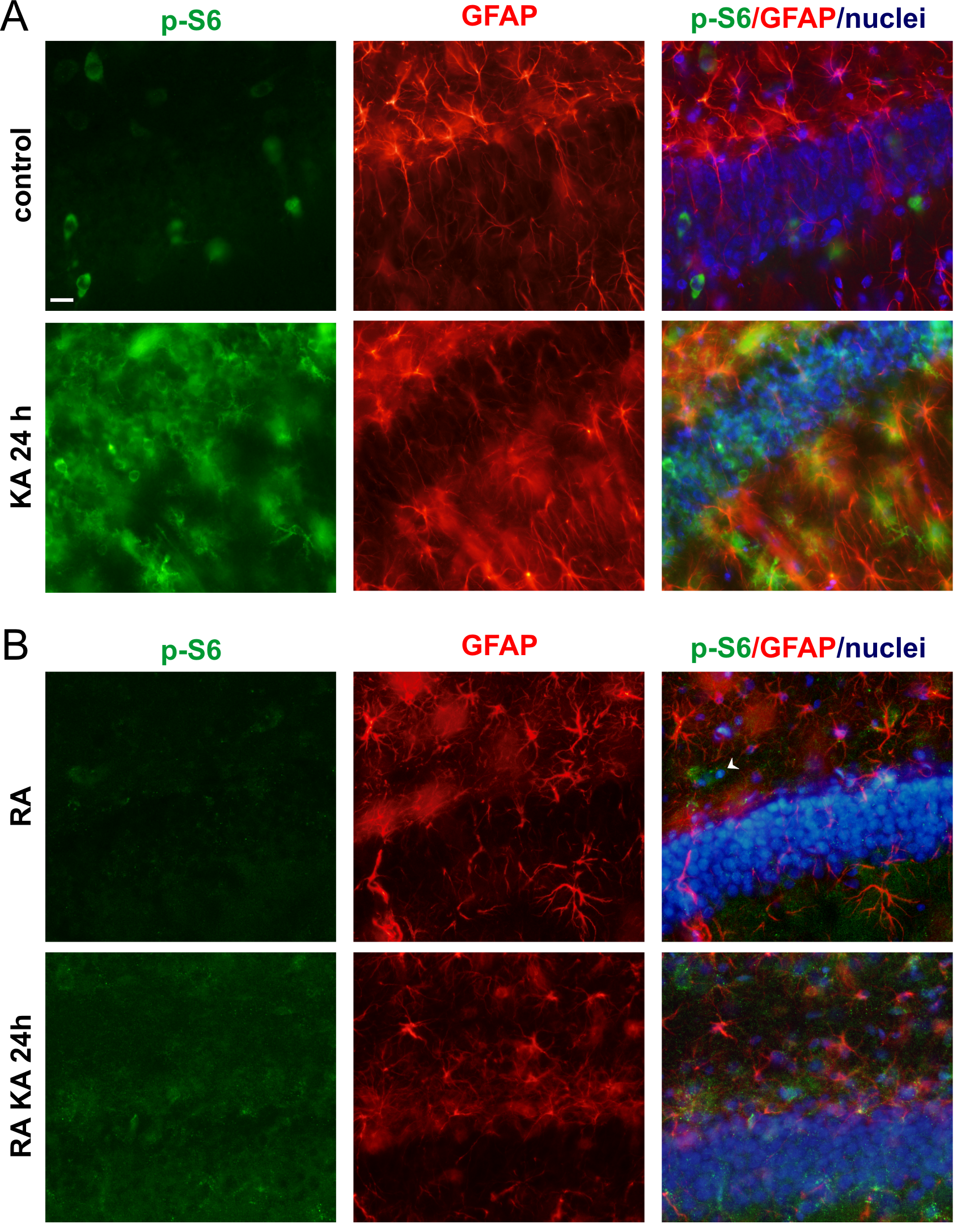

Supplement: Figure S3 — Kainic acid induces and rapamycin prevents increase in GFAP expression and in P-S6 phosphorylation in astrocytes. ( A ) Representative of dentate gyrus sections immunofluorescently stained for P-S6 (green) and astrocytic marker GFAP (red) in control animals and in animals that received kainic acid (KA) treatment and were evaluated at 2 and 24 h. ( B ) Representative images of DG sections immunofluorescently stained for P-S6 (green) and astrocytic marker GFAP (red) in chronically rapamycin (RA)- and RA+KA-treated animals 2 and 24 h after KA treatment. Scale bar = 20 µm. (TIF) [file pone.0064455.s003.tif]

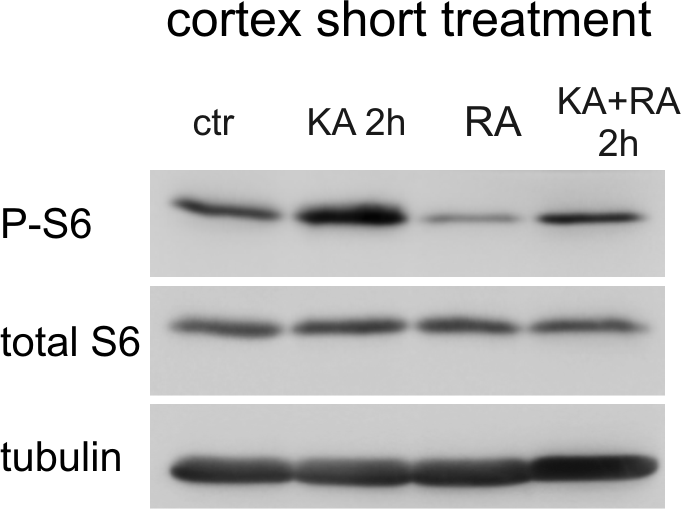

Supplement: Figure S4 — One week treatment with rapamycin lowers basal and KA-induced phosphorylation of rpS6 at Ser235/236 in rat cortex. Western blot analysis of phosphorylated rpS6 (P-S6) levels in the cortices in control (Ctr) animals, animals that received kainic acid (KA) treatment and were evaluated at 2 h, animals that received rapamycin (RA) treatment for 1 week, and animals that received both KA and rapamycin treatment. (TIF) [file pone.0064455.s004.tif]

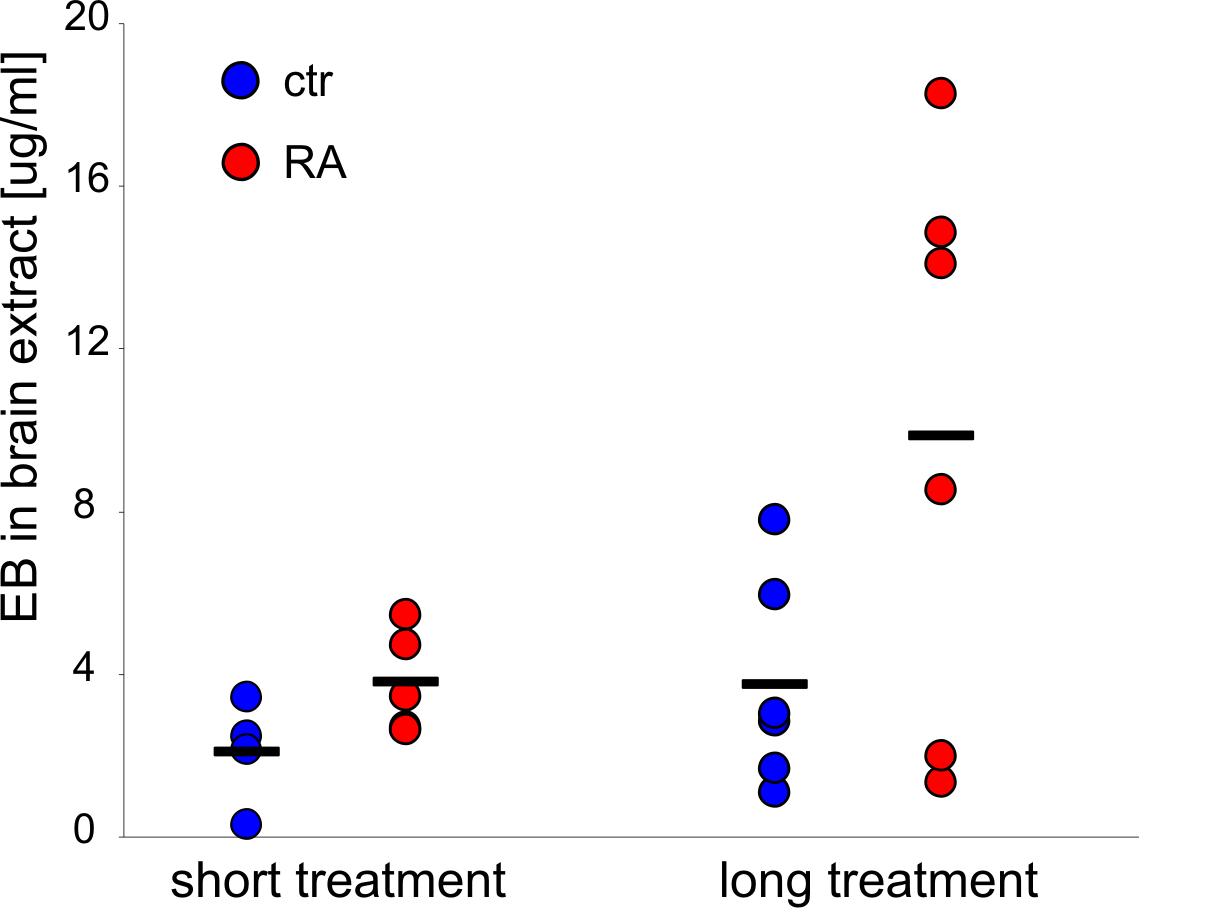

Supplement: Figure S5 — Analysis of effects of chronic rapamycin (RA) treatment on permeability of blood brain barrier with Evans Blue (EB) dye. Quantification of absorbance of supernatants obtained from brain lysates of control and rapamycin treated rats after trichloric acid precipitation (see Materials and Methods for details). Short treatment = 1 week rapamycin treatment (3 doses per week). Long treatment = 4 weeks rapamycin (3 doses per week). Control animals received vehicle. (TIF) [file pone.0064455.s005.tif]
